# Targeting IGF1‐Induced Cellular Senescence to Rejuvenate Hair Follicle Aging

**DOI:** 10.1111/acel.70053

**Published:** 2025-03-30

**Authors:** Yang Wang, Jian Yang, Yue Luo, Zhiqiang Zhao, Yawen Yuan, Juan Li, Yang Liu, Yong Yi, Xiaoke Xu, Yuankunyu Lan, Juan Zou, Qintong Li, Liang Wang, Yang Pan, Yuanhan Yang, Muzhao Xiong, Min Wu, Jinsong Li, Weiyuxin Li, Yujun Zhang, Yang Cao, Yi Zhu, Zhi‐Xiong Jim Xiao

**Affiliations:** ^1^ Center of Growth, Metabolism and Aging, Key Laboratory of Bio‐Resource and Eco‐Environment, Ministry of Education, College of Life Sciences Sichuan University Chengdu China; ^2^ State Key Laboratory of Biotherapy West China Hospital, Sichuan University Chengdu China; ^3^ Department of Pathology, West China Second University Hospital, Key Laboratory of Birth Defects and Related Diseases of Women and Children, Ministry of Education Sichuan University Chengdu China; ^4^ Departments of Obstetrics & Gynecology and Pediatrics, West China Second University Hospital, Key Laboratory of Birth Defects and Related Diseases of Women and Children, Ministry of Education, Development and Related Diseases of Women and Children Key Laboratory of Sichuan Province Sichuan University Chengdu Sichuan China; ^5^ Department of Physiology and Biomedical Engineering, Robert & Kogod Center on Aging Mayo Clinic Rochester Minnesota USA

**Keywords:** cellular senescence, dietary restriction, hair follicles, IGF‐1, p53, senolytics, SIRT1

## Abstract

The insulin‐like growth factor‐1 (IGF‐1) signaling pathway is known as a potent aging modifier, disruption of which consistently associates with lifespan extension across diverse species. Despite this established association, the mechanisms by which IGF‐1 signaling modulates organ aging remain poorly understood. In this study, we assessed age‐related changes in IGF‐1 expression across multiple organs in mice and identified a more prominent increase in skin IGF‐1 levels with aging—a phenomenon also observed in human skin. To explore the consequences of elevated IGF‐1, we developed transgenic mice ectopically expressing human IGF‐1 in the epidermis, driven by the bovine keratin 5 promoter (IGF‐1 Tg). These mice exhibited premature aging of hair follicles, as evidenced by accelerated hair graying and loss. Single‐cell RNA sequencing analyses of dorsal skin highlighted an upsurge in cellular senescence markers and the senescence‐associated secretory phenotype (SASP) in hair follicle stem cells (HFSCs), alongside a decline in hair growth and HFSC exhaustion. Our findings indicate that excessive IGF‐1 triggers HFSC senescence, thereby disrupting hair follicle homeostasis. Remarkably, interventions in IGF‐1 signaling via downstream mechanisms—specifically blocking Ac‐p53 activation via SIRT1 overexpression or senolytic treatment for senescent cell clearance, or reducing IGF‐1 through dietary restriction—significantly reduced senescence markers, mitigated premature hair follicle aging phenotypes, and restored the stem cell pool. Our findings provide fundamental insights into the biological processes of hair aging and highlight the therapeutic promise of targeted interventions to rejuvenate aged HFSCs and promote hair follicle health.

## Graphical abstract

Age‐related increases in IGF‐1 expression in the skin accelerate stem cell exhaustion and senescence by impairing SIRT1 function, ultimately driving hair follicle aging. Conversely, activating SIRT1, clearing senescent cells, or adopting dietary restriction can mitigate excessive IGF‐1 signaling and provide a promising strategy to rejuvenate hair follicle stem cells.

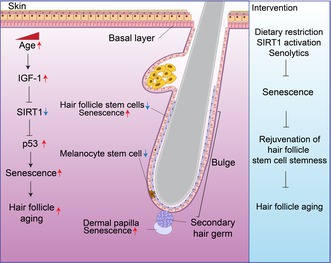

## 1 Introduction

The IGF‐1 signaling pathway, evolutionarily conserved across diverse species such as *C. elegans*, *Drosophila melanogaster* , rodents, and humans, is essential for regulating diverse important biological processes, particularly in aging (Russell and Kahn 2007; Barbieri et al. 2003). Mounting evidence implies that high levels of IGF‐1 in older adults may elevate their risk of age‐related chronic diseases and mortality, while attenuated IGF‐1 signaling has been shown to extend lifespan in several model systems (Russell and Kahn 2007; Rahmani et al. 2022; Mukama et al. 2023; Vitale et al. 2019; Mao et al. 2018). Despite its well‐documented effects on aging, the precise connection between IGF‐1 signaling and the aging processes remains complex and largely unresolved. Critical uncertainties include the specific cellular and molecular mechanisms by which IGF‐1 affects aging in various tissues and organ systems, the downstream pathways that mediate these effects, and how modulation of IGF‐1 signaling affects not only longevity but also healthspan. These gaps highlight the need for further investigations to elucidate the detailed role of IGF‐1 in aging and its potential therapeutic implications.

Our previous study and others showed that prolonged IGF‐1 treatment suppresses SIRT1 deacetylase activity. This suppression leads to increased acetylation and subsequent activation of p53, ultimately inducing cellular senescence in cultured cells (Tran et al. 2014; Nishizawa et al. 2016; Zhao et al. 2020). Cellular senescence, recognized as a hallmark of aging, plays a key role in the progression of aging and age‐related diseases (López‐Otín et al. 2023). Senescent cells accumulate in various tissues with aging, expressing pathogenic senescence‐associated secretory phenotype (SASP) that affects both nearby and distant cells and contributes to the aging processes (Zhang et al. 2022; Gorgoulis et al. 2019). However, the impact of IGF‐1‐induced cellular senescence on organismal aging remains unclear.

Hair follicles, intricate structures extending from the epidermis into the dermis, exhibit graying and loss as key visual signs of aging (Matsumura et al. 2016; Steingrimsson et al. 2005). These changes largely result from the depletion and functional decline of hair follicle stem cells (HFSCs) and melanocyte stem cells (MSCs), accompanied by prolonged dormancy. Melanogenesis, which drives hair pigmentation, is closely linked with the hair regeneration cycle and the renewal and differentiation of MSCs (Nishimura et al. 2002). The regeneration of HFSCs is essential for maintaining MSC populations, which are critical during the hair cycle for the renewal and preservation of hair pigment (Tanimura et al. 2011). Age‐related increases in stem cell senescence within hair follicles are posited to lead to a decline in MSCs, thereby contributing to hair graying (Iida et al. 2020).

In this study, we revealed a pronounced age‐related increase in IGF‐1 expression predominantly in the skin compared to other organs. Overexpression of IGF‐1 in the skin induced stem cell senescence and exhaustion, accelerating hair follicle aging in mice. Our results show that excessive IGF‐1 triggers a cascade from cellular senescence to tissue‐level aging, disrupting hair follicle homeostasis. Intervening in the downstream pathways of IGF‐1 signaling effectively reduced senescence markers, alleviated premature aging phenotypes in hair follicles, and restored the functionality of HFSCs. Collectively, our findings establish critical connections between IGF‐1‐induced cellular senescence and organismal aging, identifying potential targets for interventions to improve and rejuvenate tissue health.

## 2 Results

### 2.1 Elevated IGF‐1 Levels in the Epidermis Enhance Cellular Senescence and Accelerate Aging in Hair Follicles

**FIGURE 1:**
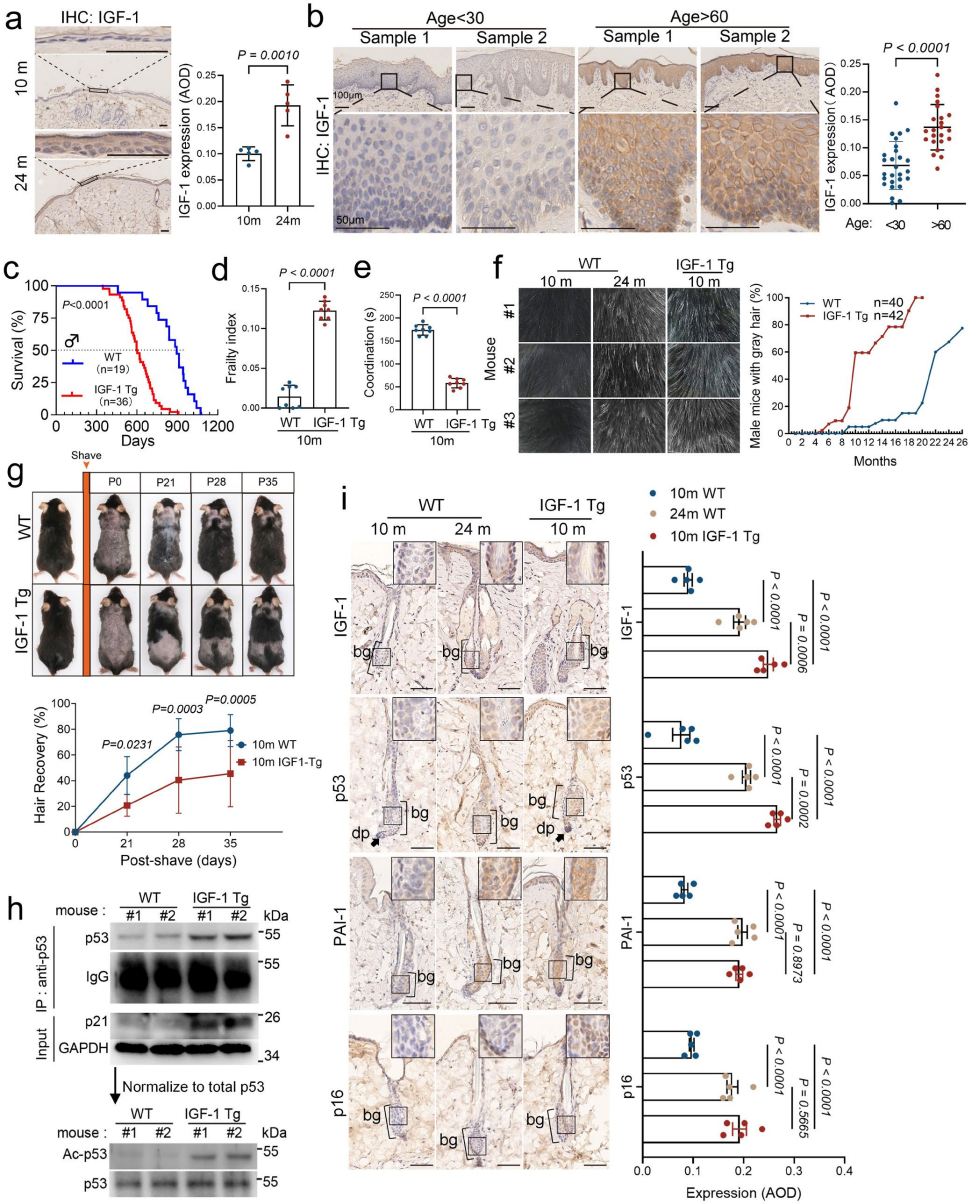
Elevated IGF‐1 expression accelerates hair follicle aging through p53‐dependent cellular senescence. (a) Protein expressions of IGF‐1 in mouse skin are assessed by immunohistochemistry (IHC) and show a significant increase in aged mice. Representative staining and average optical density (AOD) quantification were demonstrated. Data are presented as means ± SEM, Two‐tailed Student's *t*‐test. (b) IHC was performed on human perineal skin to measure IGF‐1 expression in two age groups: Younger women (< 30 years) and older women (> 60 years). Representative staining and AOD quantification were demonstrated. AOD analysis was performed on a minimum of 200 cells from the dorsal skin. Data are presented as means ± SEM, Two‐tailed Student's *t*‐test. (c) Kaplan–Meier plot shows the lifespan follow‐up in male IGF‐1 Tg mice (*n* = 36, red) or WT littermates (*n* = 19, blue). *p* values were determined by the log‐rank (Mantel‐Cox) test. (d and e) Clinical frailty index scores and rotarod test of 10‐month WT and IGF‐1 Tg male mice (*n* = 8/group). Data were means ± SEM. Statistical analysis was performed using a two‐tailed Student's *t*‐test. (f) Representative images of dorsal skin coat color from 10‐month‐old IGF‐1 Tg mice, 10‐month‐old WT mice, and 24‐month‐old WT mice are shown. The age‐dependent appearance of gray hair in mice is graphed and quantified. (g) 10‐month‐old IGF‐1 Tg male mice and age‐matched WT male mice were shaved and monitored for hair coat recovery. Hair regrowth was quantified as the percentage of back skin covered by new hairs. Data are shown as means ± SD, *n* = 5/group. Two‐tailed Student's *t*‐test. (h) Equal amounts of total tissue lysates from the dorsal skin of two 10‐month‐old IGF‐1 Tg male mice and their WT littermates were immunoprecipitated and analyzed by Western blotting to detect p53 and p21. The immunoprecipitated lysates were normalized for total p53, and the expression level of acetylated p53 at lysine 379 (Ac‐K379‐p53) was quantified. (i) Histological examination of dorsal skin was performed on 10‐month‐old IGF‐1 Tg mice, 10‐month‐old WT mice, and 24‐month‐old WT mice. Representative IHC images and quantitative analyses of IGF‐1, p53, p16, and PAI‐1 were presented. Data are shown as means ± SEM, *n* = 5/group. Two‐way ANOVA with Tukey's test. Scale bar = 50 μm.

To explore the age‐related effects of elevated IGF‐1, we initially analyzed its mRNA expression across multiple tissues at different ages using data from the Tabula Muris Senis database. This analysis revealed a notable age‐dependent increase in IGF‐1 expression predominantly in mouse skin (Figure S1a). We further examined IGF‐1 protein expression in mouse and human skin using immunohistochemistry (IHC). We observed an elevation of IGF‐1 levels in naturally aged mice (24‐month‐old) (Figure 1a). Similarly, in human skin, there was a significant accumulation of IGF‐1 in the epidermal cells of perineal skin from older women (age > 60 years), compared to lower levels observed in younger women (age < 30 years) (Figure 1b).

Next, we examined the effects of excess IGF‐1 on the skin. We developed transgenic mice (IGF‐1 Tg) that specifically overexpress the human IGF‐1 (hIGF‐1) in the epidermis under the control of the bovine keratin 5 promoter (BK5) in the C57BL/6 strain (Figure S2a,b). As shown in Figure S2c,d, the expression of IGF‐1 was significantly increased in the skin, prostate, and lung of the IGF‐1 Tg mice, while a small but significant increase of IGF‐1 was observed in the kidney, liver, and intestine. By contrast, little detectable IGF‐1 expression was noticed in muscles, heart, spleen, or testis. In addition, ELISA assays showed significantly elevated levels of exogenous human IGF‐1 (hIGF‐1) in the serum of IGF‐1 Tg mice, reaching approximately 300 ng/mL, yet there was no significant alteration of the endogenous levels of mIGF‐1, GH, or insulin in 2‐ or 10‐month WT or IGF‐1 Tg mice (Figure S2e,f). We further examined IGF‐1 Tg mice for age‐associated phenotypes, including lifespan, body weight, frailty (Liu et al. 2014; Whitehead et al. 2014), physical activity (rotarod), and coat color. As shown in Figure S3a, IGF‐1 Tg mice exhibited little difference in age‐associated body weight compared to the WT littermates. Strikingly, IGF‐1 Tg mice exhibited significantly shortened lifespans (Figure 1c) and displayed multi‐levels of premature age‐associated phenotypes, including high aging‐associated frailty index scores and deterioration in coordination (Figure 1d,e).

Further study of the IGF‐1 Tg mice showed premature hair aging, exhibiting graying and hair loss by 10 months, a phenotype typically observed in naturally aged mice at 24 months (Figure 1f and Figure S3b). Notably, accelerated hair graying was apparent in IGF‐1 Tg mice as early as 6 months, with over half of these mice displaying gray hair by the 10‐month mark (Figure 1f). Additionally, both 2‐ and 10‐month‐old IGF‐1 Tg mice exhibited slower hair regrowth post‐shaving compared to their WT counterparts, suggesting impaired hair cycle progression with delayed anagen initiation and prolonged telogen phase (Figure 1g and Figure S3c,d). To investigate the functional and molecular alterations, we performed bulk RNA sequencing on skin tissues from 10‐month‐old IGF‐1 Tg mice, age‐matched WT mice, and naturally aged WT mice (24‐month‐old). Gene set enrichment analyses (GSEA) revealed that the skin transcriptome of 10‐month‐old IGF‐1 Tg mice closely resembled that of naturally aged mice across major skin cell types (Figure S4a). Specifically, pathways associated with hair follicle development and growth, including hair follicle maturation, hair cycle activity, and stem cell differentiation, were similar in 10‐month‐old IGF‐1 Tg mice to those observed in naturally aged mice (Figure S4b). These similarities suggest that the elevated expression of IGF‐1 accelerates hair follicle aging by disrupting hair follicle homeostasis.

We have previously shown that prolonged IGF‐1 exposure induces premature cellular senescence via the SIRT1‐p53 axis in cultured cells (Tran et al. 2014). This process involves a reduction in SIRT1 deacetylase activity, leading to hyperactivation of p53 through increased acetylation at K382‐p53 (Ac‐K382‐p53, mouse Ac‐K379‐p53) (Tran et al. 2014). Based on these observations, we analyzed p53 and Ac‐K379‐p53 levels using western blotting in skin lysates from two 10‐month‐old male IGF‐1 Tg mice and their wild‐type (WT) littermates. The results confirmed increased total p53 and Ac‐K379‐p53 expression in the IGF‐1 Tg mice (Figure 1h). We further assessed cellular senescence in the epidermis and hair follicles of IGF‐1 Tg mice, comparing it to both age‐matched WT mice and naturally aged WT mice (24 months). As shown in Figure 1i and Figure S4c, the elevated levels of either IGF‐1, p53, the senescence marker p16, or a SASP marker PAI‐1 were observed in the IGF‐1 Tg mice relative to 10‐month‐old WT mice. Notably, the expression of these markers was apparently higher than that in the 24‐month‐old WT mice, indicating an accelerated senescence phenotype in IGF‐1 Tg mice and supporting our previous findings that hyperactivation of p53 driven by IGF‐1 promotes cellular senescence.

### 2.2 Single‐Cell RNA Sequencing Identifies Cellular Senescence in Mediating IGF‐1‐Driven Hair Follicle Aging

**FIGURE 2:**
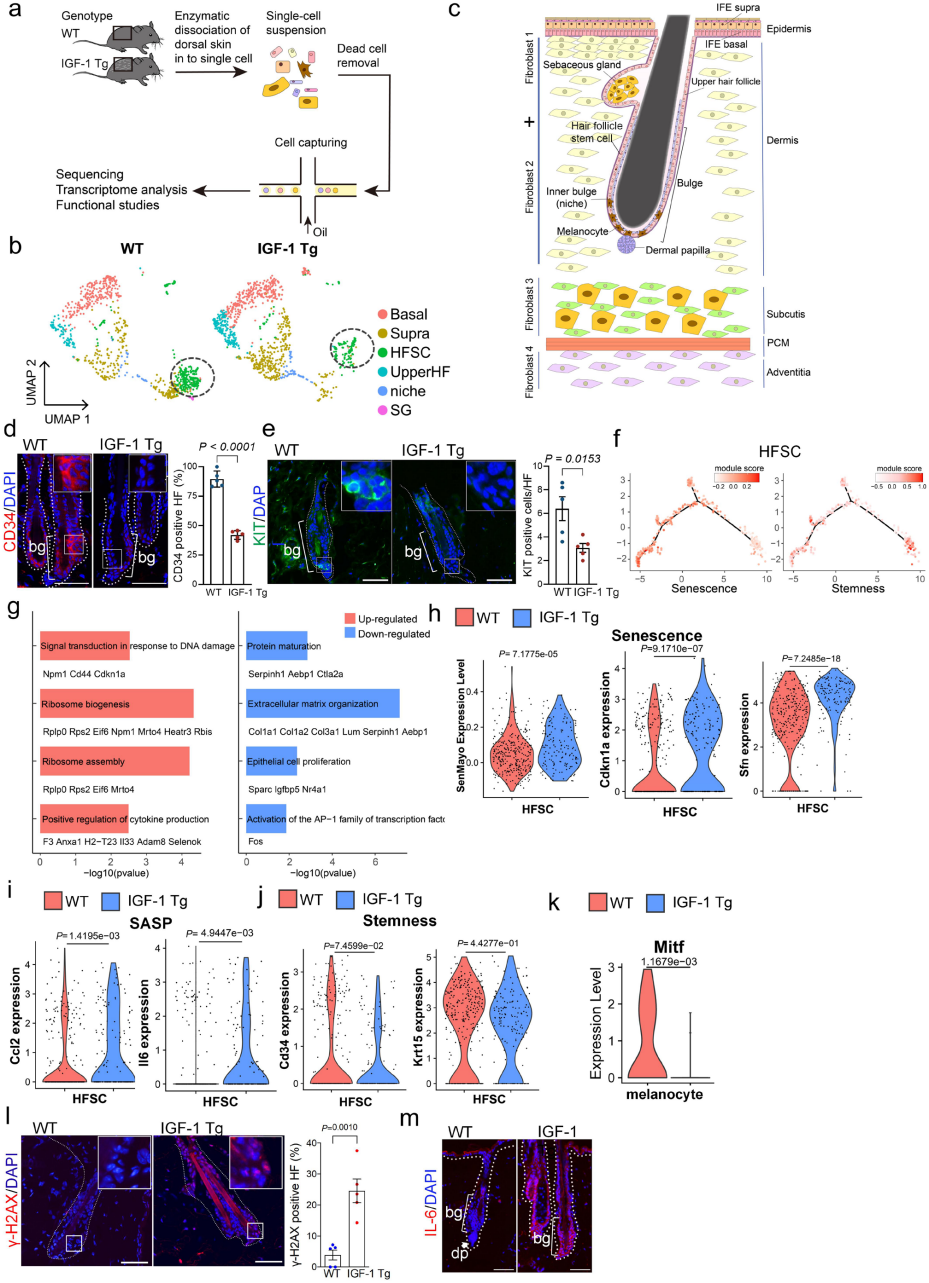
Single‐cell RNA sequencing reveals IGF‐1‐induced cellular landscapes and aging profiles in mouse skin. (a) Schematic overview of the single‐cell RNA sequencing experiment conducted on mouse skin. (b) Uniform manifold approximation and projection (UMAP) plots depicting the distribution of six epidermal cell types in the skin of wild‐type and IGF‐1 transgenic (Tg) mice. Major cell types are classified using marker genes and are color‐coded according to cell identity: Basal, HFSC, Niche, and Upper HF region. (c) Structural and Cellular Composition of Mouse Skin. Interfollicular epidermis is abbreviated to IFE. Panniculus Carnosus Muscle is abbreviated to PCM. Figure modified based on *Cell Stem Cell*, 2020 (Joost et al. 2020). (d and e) Representative IF images and corresponding quantitative analyses are shown for (d) hair follicle stem cell (HFSC) marker CD34 (*n* = 5/group) and (e) melanocyte stem cell marker KIT (*n* = 5/group). (f) Module scores, based on genes related to cell senescence and stemness, are plotted along the pseudotime trajectory of HFSC cells. Color intensity indicates the enrichment of scores associated with cellular senescence or stemness. (g) Bar plot illustrating the enriched Gene Ontology and Reactome terms for upregulated and downregulated genes in HFSCs comparing WT and IGF‐1 Tg mice. (h–k) Violin plots illustrate the differences in module scores between HFSCs from IGF‐1 Tg mice and age‐matched WT mice, calculated using genes from the SenMayo gene set, Student's *t*‐test (h). Violin plots display expression differences for genes related to HFSC senescence (*Cdkn1a* and *Sfn*) (h), SASP (*Ccl2* and *Il6*) (i), and stemness (*Cd34* and *Krt15*) (j). The expression of *Mitf* in melanocytes (k) was analyzed using the Wilcoxon rank‐sum test. (l and m) Representative IF images and corresponding quantitative analyses of the senescence‐associated DNA damage marker γ‐H2AX and the SASP factor IL‐6 (*n* = 5/group).

To comprehensively understand how IGF‐1 promotes hair follicle aging, we harvested mouse epidermis from 10‐month‐old IGF‐1 Tg mice and 10‐month‐old WT mice and performed scRNA‐seq using the 10× droplet‐based method (Figure 2a). We retrieved a total of 14,439 cells from IGF‐1 Tg and WT age‐matched mice. Using the Seurat package (Hao et al. 2021), we identified five major cell types—epidermis, fibroblasts, immune cells, vascular cells, and neural‐crest‐derived cells—each distinguished by unique cellular markers (Figure S5a,b). Notably, the BK5 promoter, which drives human IGF‐1 expression in the IGF‐1 Tg mouse model, was exclusively active in epidermal cells, suggesting the targeted action of IGF‐1 within the epidermis (Figure S5a). Therefore, based on their unique transcriptomic signatures (Zhang et al. 2021) (Figure 2b and Figure S4c, schematic illustration in Figure 2c), epidermal cells could further be characterized in five distinct sub‐clusters, including basal cells, suprabasal cells, HFSCs, upper hair follicle cells (upper HF, uHF), and niche cells. Further analyses revealed a marked decrease in the HFSCs pool, which was verified using immunofluorescence staining (IF) for the expression of the HFSC marker CD34 or the melanocyte stem cell marker KIT in the HF bulge region (Figure 2d,e). Concomitantly, an increase in differentiated upper HF cells in IGF‐1 Tg mice was noticed (Figure 2b and Figure S5d,e), suggesting a depletion of HFSCs (Figure S5e).

As shown in Figure 2f, module scores, derived from genes associated with cellular senescence and stem cell stemness, were mapped onto a pseudotime trajectory using HFSC data. These analyses revealed that HFSCs with high stemness scores and those with high senescence scores followed distinct developmental trajectories, which were mutually exclusive, suggesting a progression from stemness to senescence. Furthermore, the differential expression genes of HFSCs in IGF‐1 Tg mice compared to WT controls showed that upregulated genes were predominantly involved in DNA damage response, cytokine production, and ribosome biogenesis pathways, whereas downregulated genes were linked to cell proliferation and protein maturation (Figure 2g), suggesting a strong association with cellular senescence in HFSCs of IGF‐1 Tg mice. Moreover, module scores for senescence‐associated gene sets (SenMayo) were significantly higher in HFSCs from IGF‐1 Tg mice than those from age‐matched WT mice (Figure 2h). Expression levels of senescence markers, such as p21/*Cdkn1a* and 14‐3‐3σ/*Sfn*, and SASP components, including *Ccl2* and *Il6*, were markedly increased (Figure 2h,i). In contrast, markers of stemness, such as *Cd34* and *Krt15*, were decreased (Figure 2j). Additionally, the expression of *Jag1* and *Tgfb1*, both crucial for MSC homeostasis (Nishimura et al. 2010), was significantly decreased (Figure S5f). Together, these results suggest that increased cellular senescence is closely associated with the reduction of HFSC stemness in IGF‐1 Tg mice.

The color of hair is critically dictated by the levels of melanin produced by melanocytes and then transferred to hair (Centeno et al. 2023). The transcription factor MITF (microphthalmia‐associated transcription factor) plays a critical role in melanocytes, regulating genes essential for melanin production, melanocyte survival, and differentiation. Our single‐cell RNA sequencing analyses identified distinct melanocyte clusters and revealed a significant decrease in MITF expression in melanocytes from IGF‐1 Tg mice (Figure 2k and Figure S6). Notably, elevated expression of p53, senescence markers (p16, γ‐H2AX) and SASP markers (PAI‐1 and IL‐6) were significantly elevated in the HF bulge region of 10‐month‐old IGF‐1 Tg mice compared to age‐matched WT littermates (Figures 1g, 2l,m). Importantly, while apoptosis has been linked to hair aging (Ahn et al. 2023; Adav and Ng 2023), little apoptosis was detected in hair follicles in 10‐month‐old IGF‐1 Tg mice (Figure S7), indicating that senescence is likely the primary mechanism involved.

The dermal papilla (DP), a specialized type of fibroblast, is essential to hair follicle development, significantly contributing to hair production, growth cycling, and pigmentation. Our single‐cell RNA seq analyses revealed a reduction of DPs and fibroblast1 (FAB1) cells in IGF‐1 Tg mice, concomitant with an increase in the populations of fibroblast3 (FAB3) cells (Figure S8a–c). Further examination of gene expressions in DP revealed significant upregulation of the senescence marker p21 and SASP‐associated genes, such as IL‐6 and CXCL2 (Figure S8d) in IGF‐1 Tg mice. Subsequent SA‐β‐gal staining of the dorsal skin in IGF‐1 Tg mice revealed intensified staining in DPs, comparable to that observed in naturally age‐matched mice (Figure S8e). Furthermore, there were more SA‐β‐gal staining‐positive cells in cultured primary murine whisker DP cells within the DPs from IGF‐1 Tg mice (Figure S8f). Collagen, predominantly synthesized and secreted by dermal fibroblasts, plays a crucial role in maintaining skin strength, elasticity, and mechanical barrier function (Chambers and Vukmanovic‐Stejic 2020; Gelse et al. 2003). Our results show that the expression of the cell cycle arrest gene (*Cdkn1a*) was significantly increased, while the expression of the collagen genes (*Col1a1* and *Col1a2*) was downregulated in all fibroblast populations (Figure S9a–c). Consistently, the Masson's trichrome staining revealed a substantial reduction in dermal thickness (Figure S9d). The cell–cell communication analyses revealed that HFSCs, epidermal cells (IFE), as well as FIB1 and FIB3 fibroblast clusters, could be engaged in cross‐talking through the IGF‐1 signaling pathway in IGF‐1 Tg mouse skin (Figure S9e). Together, these results suggest that IGF‐1‐induced cellular senescence contributes causally to DP deterioration.

### 2.3 Ectopic Expression of SIRT1 Rescues IGF‐1‐Induced Cellular Senescence in HFSCs and Mitigates Premature Hair Aging in IGF‐1 Tg Mice

**FIGURE 3:**
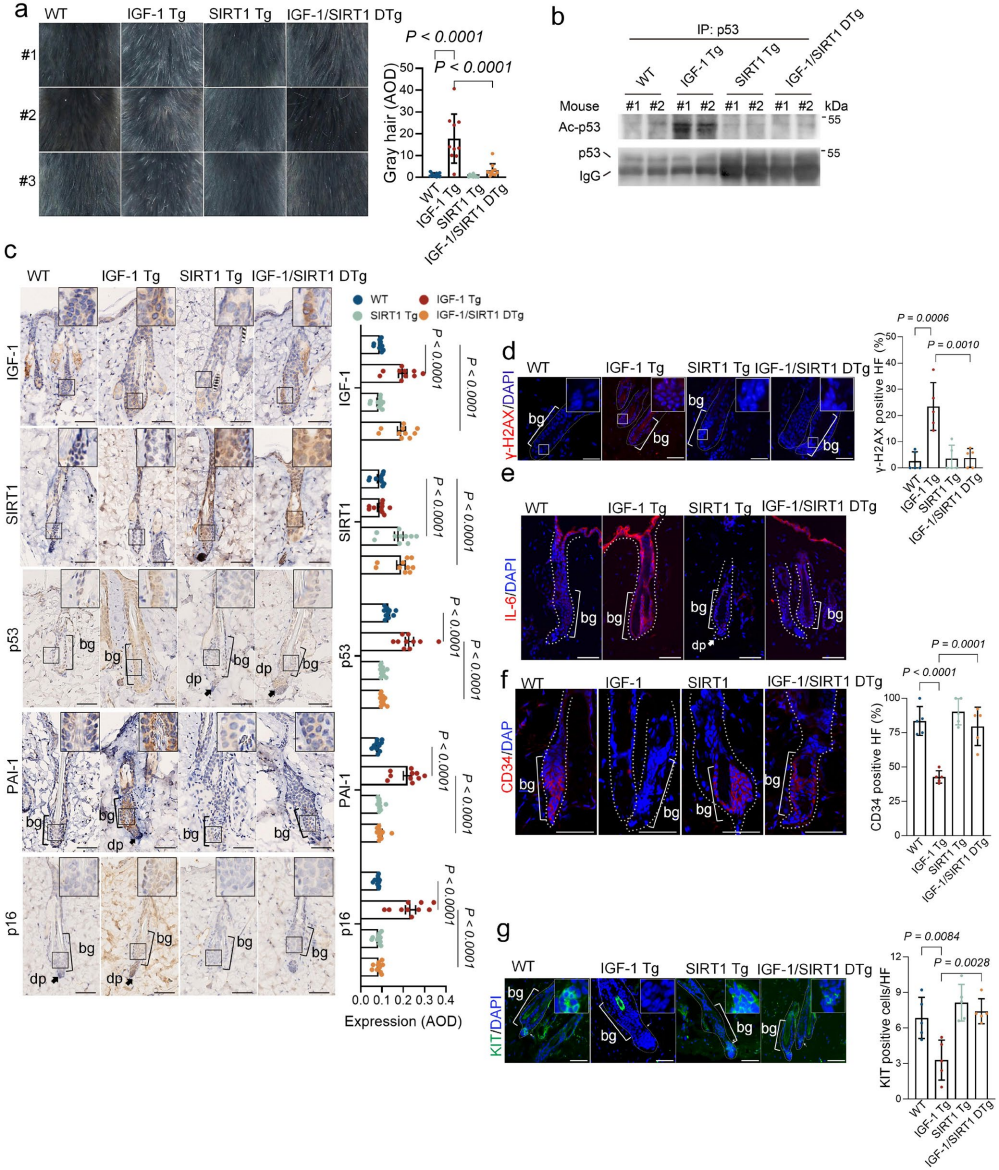
Forced SIRT1 overexpression attenuates hair graying and hair follicle cell senescence in IGF‐1 Tg mice. (a) Representative images of age‐associated coat color from 10‐month‐old male IGF‐1 Tg mice, SIRT1 Tg mice, IGF‐1/SIRT1 double transgenic (DTg) mice, and WT littermates, showing dorsal gray hair (*n* = 10/group). The percentage of mice with gray hair in each group is also presented. (b) Equal amounts of total tissue lysates from the dorsal skin of these mice were immunoprecipitated and analyzed by Western blotting with a p53‐specific antibody. The immunoprecipitated samples were normalized to total p53 levels before immunoblotting for Ac‐K379‐p53. (c) Histological examination of dorsal skin was conducted to assess IGF‐1 signaling. Representative IHC images and corresponding quantitative analyses are shown for IGF‐1, SIRT1, p53, p16, and PAI‐1 (*n* = 8/group). (d–g) Representative IF images and corresponding quantitative analyses of senescence‐associated DNA damage marker γ‐H2AX, SASP factor IL‐6, HFSC marker CD34, and melanocyte stem cell marker KIT (*n* = 5/group). Data are presented as means ± SEM; two‐way ANOVA with Tukey's test. Scale bar = 50 μm.

Our previous study demonstrates that chronic IGF‐1 exposure diminishes SIRT1 deacetylase activity, resulting in elevated acetylated p53 and, consequently, cellular senescence in vitro (Tran et al. 2014). In this study, we show that IGF‐1 Tg mice display elevation of acetylated p53, accompanied by accelerated cellular senescence in the epidermis and premature aging in the hair follicles. We hypothesized that enhancing SIRT1 function could reduce IGF‐1‐induced senescence in mitigating hair aging in early‐aged IGF‐1 Tg mice. To test this, we generated transgenic mice (SIRT1 Tg) by epithelial‐targeting expression of murine SIRT1 under the BK5 promoter in C57BL/6 mice, using a method that was utilized for IGF1‐Tg mice (Figure S10a). As shown in Figure S10b, expression of SIRT1 was significantly higher in the skin compared to the liver and kidney, similar to what was observed in IGF‐1 Tg mice. To investigate whether SIRT1 overexpression can rescue IGF‐1‐induced hair follicle aging, IGF‐1 Tg mice were crossed with SIRT1 Tg mice to generate IGF‐1/SIRT1 double transgenic (DTg) mice (Figure S10c). We then assessed the dorsal coat color of male IGF‐1/SIRT1 double transgenic mice (IGF‐1/SIRT1 DTg) at 10 months of age, comparing them to either WT littermates or single transgenic mice (IGF‐1 Tg or SIRT1 Tg). In contrast to most IGF‐1 Tg mice that exhibited hair graying, the IGF‐1/SIRT1 DTg mice, along with WT and SIRT1 Tg mice, displayed normal coat color (Figure 3a).

Notably, the IGF‐1‐induced upregulation of acetylated K379‐p53 was completely inhibited in the dorsal skin of the IGF‐1/SIRT1 DTg mice (Figure 3b). Furthermore, expression of the senescence markers, including p53, p16, PAI‐1, γ‐H2AX, and IL‐6 in the HF bulge region of the IGF‐1/SIRT1 DTg mice was comparable to that in age‐matched WT or SIRT1 Tg mice, but significantly lower than that observed in IGF‐1 Tg mice (Figure 3c). Concurrently, the expression of stemness markers, CD34 and KIT, was elevated in IGF‐1/SIRT1 DTg mice relative to IGF‐1 Tg mice (Figure 3d–g).

### 2.4 Clearance of Senescent Cells by Senolytics Rejuvenates Hair Follicle Stem Cells and Mitigates Hair Graying

**FIGURE 4:**
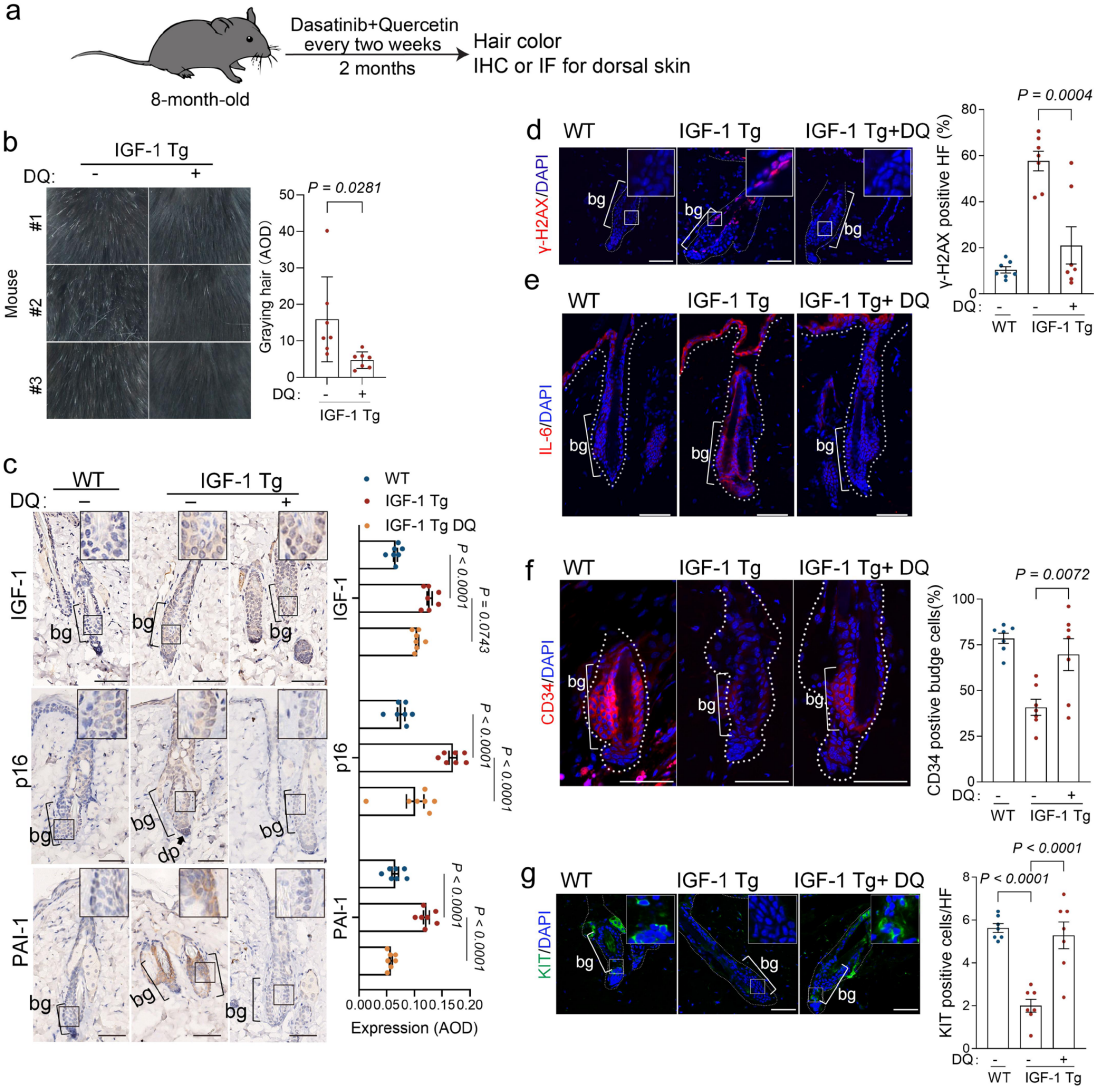
Senolytics alleviate hair follicle aging phenotypes in IGF‐1Tg mice. (a) Experimental design for senolytic treatment in IGF‐1Tg mice. 8‐month‐old male IGF‐1 Tg and WT littermates were administered senolytics (D + Q) via oral gavage for 2 months (*n* = 7/group). Age‐associated coat color and histological examinations were performed. (b) Representative coat color images and AOD quantification are shown. (c) Representative IHC images and corresponding quantification of IGF‐1, p16, and PAI‐1. (d–g) Representative IF images and quantification of the DNA damage marker γ‐H2AX (d), IL‐6 (e), CD34 (f), and KIT (g) are presented as means ± SEM; two‐way ANOVA with Tukey's test. Scale bar = 50 μm.

We next investigated whether the deleterious effects induced by IGF‐1 could be mitigated through the clearance of senescent cells using senolytics. We administered a treatment regimen to 8‐month‐old IGF‐1 Tg mice, with either a vehicle or a senolytic combination of dasatinib and quercetin (DQ) biweekly for two months (Whitehead et al. 2014), followed by an assessment of the coat color and senescence markers using IHC or IF (Figure 4a). Notably, the senolytics led to a significantly reduced gray hair in the dorsal region (Figure 4b). Additionally, senolytics did not significantly alter IGF‐1 levels (Figure 4c). However, it significantly reduced the expression of senescence markers (p16 and γ‐H2AX) and SASP components (PAI1 and IL‐6) (Figure 4c–e). These results suggest that senolytics effectively targeted and eliminated senescent cells in IGF‐1 Tg mice. Remarkably, this intervention could also significantly increase CD34^+^ HFs and KIT^+^ MSCs, a sign of rejuvenation for the stem cell pool (Figure 4f,g).

### 2.5 DR Modulates IGF‐1 Signaling to Mitigate HFSC Senescence and Hair Follicle Aging in IGF‐1 Tg Mice

**FIGURE 5:**
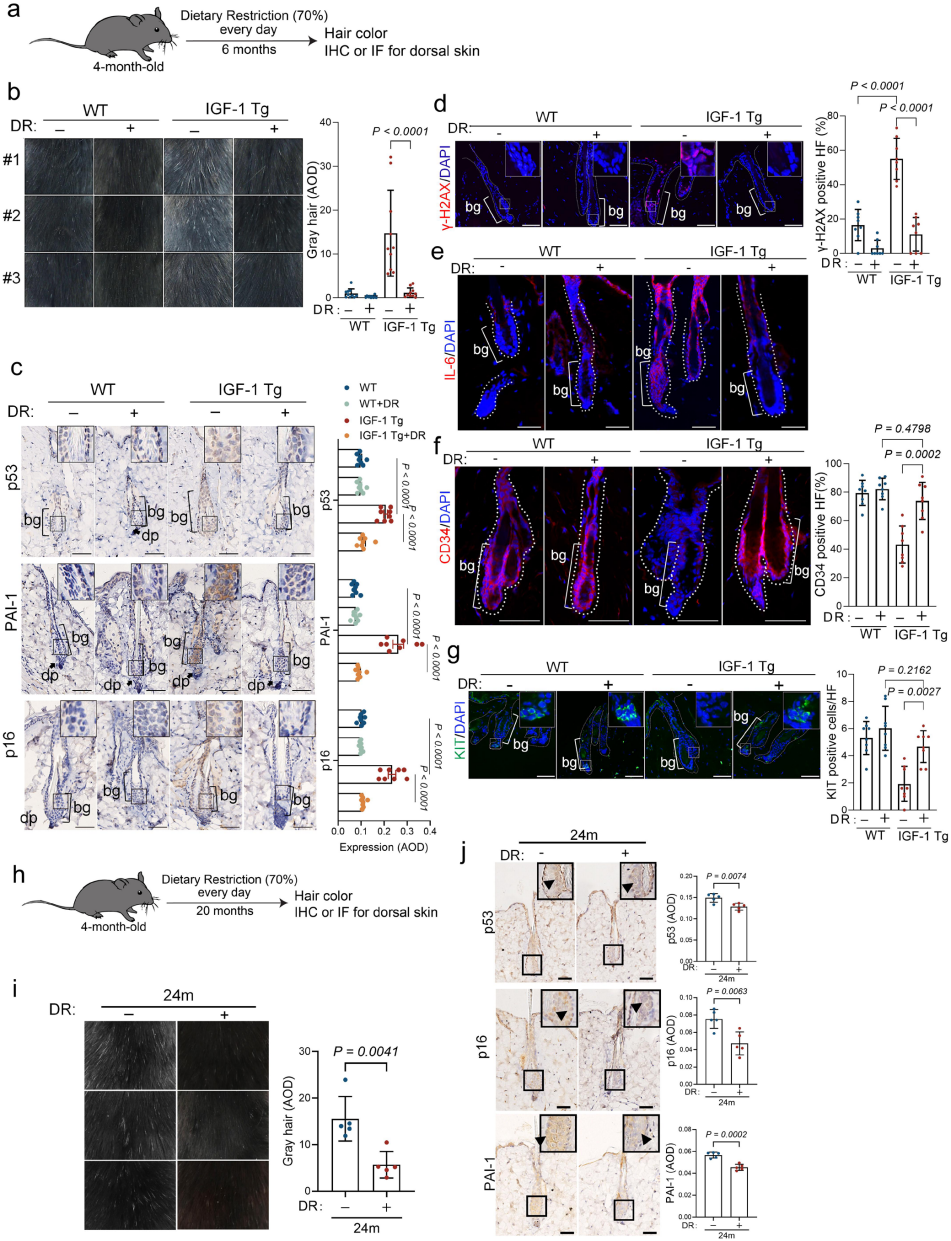
Dietary Restriction targeting IGF‐1 alleviates hair graying and HFSC senescence. (a) Four‐month‐old male IGF‐1 Tg mice and WT littermates were fed either regular chow or subjected to a 30% dietary restriction (*n* = 11/group) for 6 months. Dorsal coat color and hair graying were assessed, followed by IHC and IF analyses of hair follicles. (b) Representative images of dorsal coat color and percentages of mice with gray hair in each group were analyzed. (c) Representative IHC images and corresponding quantitative analyses of IGF‐1, SIRT1, p53, p16, and PAI‐1 (*n* = 7/group). (d–g) Representative IF images and corresponding quantitative analyses of the senescence‐associated DNA damage marker γ‐H2AX (d). SASP factor IL‐6 (e), hair follicle stem cell marker CD34 (f), and melanocyte stem cell marker KIT (g) (*n* = 5/group). Data were means ± SEM, Two‐way ANOVA with Tukey's test. Scale bar = 50 μm. (h–j) 4‐month‐old male IGF‐1 Tg mice and WT littermates were fed either regular chow or subjected to a 30% dietary restriction (*n* = 5/group) for 20 months. Dorsal coat color and hair graying were assessed, followed by IHC and IF analyses of hair follicles (h). Representative coat color images and AOD quantification are shown (*n* = 5/group) (i). Representative IHC images and corresponding quantitative analyses of p53, p16, and PAI‐1 (*n* = 5/group) (j).

DR is recognized for its ability to modulate IGF‐1 signaling (Sonntag et al. 1992; Breese et al. 1991; Fontana et al. 2008; Houthoofd et al. 2003), which could potentially mitigate aging processes, highlighting DR as a viable therapeutic strategy for age‐related disorders. We therefore investigated the extent to which DR impacts IGF‐1‐mediated cellular senescence and aging in the epidermis. IGF‐1 Tg mice and their WT littermates were subjected to either a normal diet or a calorie‐restricted diet (70% of normal intake) starting at 4 months of age and continuing for 6 months, followed by an assessment of HFSC senescence and hair graying (Figure 5a). As shown in Figure 5b, 10‐month‐old IGF‐1 Tg mice fed with a normal diet exhibited premature graying. In contrast, those on a calorie‐restricted diet displayed a normal coat color, comparable to that of their age‐matched WT littermates, concomitant with significantly reduced expression of p53, p16, PAI‐1, γ‐H2AX, and IL‐6 (Figure 5c–e). Notably, DR treatment rejuvenated the populations of CD34^+^ HFSCs and KIT^+^ MSCs in IGF‐1 Tg mice, comparable to those in WT littermates (Figure 5f,g). Extended DR treatment could also mitigate hair graying in naturally aged mice (Figure 5h–j).

## 3 Discussion

Epidemiological studies reveal that IGF‐1 is multifaceted and critical in aging and age‐related diseases, including cardiovascular disease (Higashi et al. 2019), type 2 diabetes (Biadgo et al. 2020), and frailty (Doi et al. 2018). Notably, elevated IGF‐1 levels in adipose tissue have been linked to metabolic disorders and an increased risk of cancer (Zhong et al. 2023). On the other hand, disrupting IGF‐1 signaling has been shown to extend lifespan across various species (Rahmani et al. 2022; Mukama et al. 2023; Vitale et al. 2019; Mao et al. 2018). A central and unsolved issue is how IGF‐1, a well‐documented growth factor, executes its function in modulating aging processes, particularly at the organ level. This study demonstrates that chronic activation of IGF‐1 signaling leads to aging‐associated senescence at the cellular and organ levels, accompanied by p53‐dependent cellular senescence, leading to hair follicle aging.

IGF‐1, a key growth signaling molecule, is known as essential for the development and maintenance of primary organs, including the brain (Huffman et al. 2016; Ashpole et al. 2015), muscles (Ascenzi et al. 2019), and blood (Florini et al. 1985). Notably, these organs show a decline in IGF‐1 levels as they age (Huffman et al. 2016; Ashpole et al. 2015; Ascenzi et al. 2019; Florini et al. 1985). However, this study reveals the age‐related increase in IGF‐1 expression in human skin specimens and in mouse skin that is associated with the accelerated aging of hair follicles. The analyses of scRNA‐seq data from the human skin aging atlas (Zou et al. 2021) also show a significant upregulation of IGF‐1 in fibroblasts in aged human skin. These observations indicate that IGF‐1 possesses tissue‐specific impacts. It is plausible that, as aging progresses, supportive organs such as fat and skin might elevate IGF‐1 production to enhance the survival of essential organs. Yet, elevated IGF‐1 in these tissues could also promote tissue aging. Indeed, our results indicate that chronic exposure to excess IGF‐1 in hair follicles results in premature hair graying and loss, primarily driven by an accumulation of senescent cells. We demonstrated that interventions targeting the IGF‐1 signaling pathway, such as overexpression of SIRT1 to inhibit p53 overactivation, senolytic clearing of senescent cells, or modulating the IGF‐1 signaling by dietary restriction (DR), effectively restore hair follicle stem cell functionality and reverse impaired hair growth and loss.

Both rodents and humans display progressive hair loss and reduced hair follicle regeneration as they age (Shin et al. 2020). Previous studies have identified cell apoptosis, triggered by accumulated DNA damage or other cellular stresses, as a key mechanism underlying the exhaustion of HFSCs and the resulting compromised hair growth (Matsumura et al. 2017; Garza et al. 2012; Shimomura et al. 2010). In this study, we provide compelling evidence that cellular senescence mediated by the IGF‐1‐p53‐SIRT1 axis significantly contributes to the exhaustion of stemness in HFSCs and is a key driver of hair loss. Our single‐cell RNA sequencing analysis demonstrated a shift in HFSCs from a state of stemness to senescence without notable apoptosis in the hair follicle. Senescent HFSCs exhibited high levels of SASP factors, including proinflammatory factors like IL‐6 and CCL2, and fibrotic factors such as PAI‐1. These SASP factors, involved in inflammation and tissue remodeling, may adversely affect HFSCs and neighboring cells via paracrine interactions. Indeed, IL‐6 has been shown to inhibit clonal growth of keratinocytes and block the transition from the telogen (resting) phase to the anagen (growth) phase of hair (Huang et al. 2017). Furthermore, normal hair follicle regeneration relies on interactions between HFSCs and the DP, with a minimum number of DP cells necessary to initiate the anagen phase of hair growth. Maintaining adequate DP cell pool is therefore essential for preserving hair follicle regenerative capacity. Our findings suggest that IGF‐1‐induced HFSC senescence, which is associated with a decrease in DP cell pool, causally impairs hair follicle regeneration.

A decline in follicular MSCs is a primary cause of hair graying (Nishimura et al. 2005). Studies in mice indicate that the MSC microenvironment, formed by HFSCs and termed the MSC niche, is crucial for MSC survival. Our findings reveal that overexpression of IGF‐1 results in the downregulation of *Jag1*, a Notch ligand, and *Tgfb1*, both crucial for MSC homeostasis (Nishimura et al. 2010). This simultaneous impairment of these signaling pathways significantly undermines the supportive niche environment, potentially leading to a reduction in MSCs. In IGF‐1 Tg mice, we also observed a marked reduction in the expression of *Mitf*, a key transcription factor essential for melanocyte development and function. This reduction appears linked to an abnormal elevation of IL‐6 in HFSCs, adversely affecting melanin synthesis, cell growth, and survival (Swope et al. 1991; Fu et al. 2020). Furthermore, these mice demonstrated increased senescence in HFSCs within hair follicles, accompanied by elevated levels of SASP, including TNFa, IL‐1, and IL‐6, which are known inhibitors of melanogenesis and could exacerbate MSC dysfunction (Fu et al. 2020). The imbalance between stem cells and differentiated cells observed in our single‐cell RNA sequencing data suggests that excessive depletion of the stem cell pool may impair hair regeneration, contributing to hair loss. This cascade of events potentially contributes to accelerated hair graying in IGF‐1 Tg mice.

DR extends lifespans across species from yeast to mice (Cohen et al. 2004; Bishop and Guarente 2007). Although DR mitigates many age‐related physiological changes in rodents, the precise mechanisms through which it influences aging remain unclear. Decades of research suggest that reduced insulin/IGF‐1 signaling might be a key mediator of DR's effects on longevity (Sonntag et al. 1992; Breese et al. 1991; Fontana et al. 2008; Houthoofd et al. 2003). Notably, one of the most consistent changes observed in rodents under DR is a reduction in IGF‐1 levels (Breese et al. 1991), indicating a potential link between this pathway and the aging process. Our study confirms that DR effectively mitigates premature aging phenotypes in hair follicles of IGF‐1 Tg mice. We show that DR not only reduces IGF‐1‐induced cellular senescence but also prevents the depletion of the stem cell pool associated with this process. Collectively, we demonstrate that DR slows the aging process through suppressing the IGF‐1‐p53 axis, underscoring that strategies aimed at reducing signaling through this pathway may be effective in promoting healthy aging.

A key finding of this study is that the IGF‐1–p53 axis plays a pivotal role in cellular senescence, driving organ aging as exemplified by hair follicle aging, and likely contributes to systemic organismal aging. This is supported by observations of shortened lifespan, increased frailty index, and impaired motor coordination in IGF‐1 Tg mice. It is plausible that IGF‐1, after being secreted locally and entering the bloodstream, exerts systemic effects on various organs. It is also possible that SASP factors, such as IL6 and CCL2, secreted by senescent hair follicles, may impair the function of other tissues, including muscles and lungs. Interestingly, while DR significantly reduces IGF‐1 expression and mitigates skin aging, downstream intervention of IGF‐1 by overexpressing SIRT1 expression in the epidermis or senolytics can also effectively alleviate hair follicle aging. These findings provide strong evidence that hIGF‐1 exerts its aging‐promoting function in the skin by inducing cellular senescence via downregulation of SIRT1.

Our initial investigations focused exclusively on the effects of IGF‐1 in male mice. Recognizing the known sexual dimorphism in IGF‐1 responses (Ashpole et al. 2017), it is possible that these effects may vary in female mice. Sexual dimorphism represents an expanding area within aging research. Further research on IGF‐1 signaling with a special emphasis on understanding sex‐specific differences as aging evolves would be desirable.

## 4 Materials and Methods

### 4.1 Western Blot, Immunofluorescence, and IHC

Western blot analyses, immunofluorescence, or IHC were performed as described (Yi et al. 2020; Wang et al. 2019; Li et al. 2022). Briefly, for western blot analyses, cells were washed twice with PBS and lysed with EBC250 buffer (250 mM NaCl, 25 mM Tris–HCl, pH 7.4, 0.5% NP‐40, and 50 mM NaF) supplemented with protease inhibitor cocktail (B14001, Selleck Chemicals). Equal amounts of total protein were fractionated by SDS/PAGE and transferred to the PVDF membrane. Non‐specific binding was blocked in 4% nonfat dry milk diluted in TBS supplemented with 0.1% Tween, and membranes were incubated with primary antibody and HRP‐conjugated secondary antibody for subsequent detection by chemiluminescence (Bio‐Rad). Images were analyzed using Image Lab Software 5.1.

For IF analyses, cells were fixed with 4% paraformaldehyde for 15 min at room temperature, permeabilized with 0.1% Triton‐100 for 15 min, blocked with 5% BSA for 1 h, and stained with specific primary antibodies followed by corresponding secondary antibodies. Nuclei were counterstained with DAPI. Images were captured using a confocal fluorescent microscope.

For IHC analyses, paraffin‐embedded samples were sliced into 5 μm thickness. Tissue sections were rehydrated through a decreasing ethanol gradient and treated by boiling in citrate buffer (pH 6.0) or Tris‐EDTA (pH 9.0) for antigen retrieval. Endogenous peroxidases were blocked using 0.3% H_2_O_2_. After blocking with 5% BSA, the sections were incubated with primary antibody and followed by horseradish peroxidase‐conjugated secondary antibody. The sections were subsequently stained with a DAB Detection Kit (ZLI‐9018, ZSGB‐BIO). For quantitative analysis, tissue slides were scanned through NanoZoomer (Hamamatsu, Japan), and the scanned images were subjected to analyzing average optical density (AOD) (Wang et al. 2019) using QuPath (Bankhead et al. 2017). The AOD value for each mouse was calculated from over 200 cells.

Antibody for SIRT1 (ab110304, WB 1:1000, IF 1:100; ab32441, WB 1:1000, IF 1:100) was purchased from Abcam (Cambridge, MA, USA). Antibodies for GAPDH (AB0037, WB 1:3000), IL‐6 (AY2682, IF 1:100), CD34 (CY5196, IF 1:100), or p21 (CY5543, WB 1:1000) were purchased from Abways Technology (Shanghai, China). Flag (F1804, WB 1:1000, IF 1:100) antibody was purchased from Sigma. Antibodies for Ac‐K379‐p53 (#2570S, WB 1:1000), γ‐H2AX (#2577, WB 1:1000, IF 1:200), and PAI‐1 (#11907, WB 1:1000, IHC 1:200) were purchased from Cell Signaling Technology (Danvers, MA, USA). Antibodies for p53 (sc‐126, WB 1:200) and mouse p16 (sc‐1207, WB 1:200) were purchased from Santa Cruz Biotech (CA, USA). Human p16 antibody (10883‐1‐AP, WB 1:1000) was purchased from Proteintech (Wuhan, Hubei, China). IGF‐1 (bs‐0014) and p16 antibodies (bs‐4592R or bs‐20656R, IHC 1:200) specific for IHC were purchased from Bioss Antibodies (Beijing, China). CD117 (Kit) monoclonal Antibody (# 14‐1172‐85) was purchased from Invitrogen. Goat anti‐mouse IgG‐HRP (sc‐2005, WB 1:2000) and goat anti‐rabbit IgG‐HRP (sc‐2004, WB 1:5000) antibodies were purchased from Santa Cruz Biotechnology (CA, USA); Rhodamine (TRITC)–conjugated AffiniPure Donkey Anti‐Rabbit IgG (711‐025‐152, IF 1:160) and Fluorescein (FITC) AffiniPure Donkey Anti‐Rabbit IgG (711‐095‐152, IF 1:160), Rhodamine (TRITC)–conjugated AffiniPure Donkey Anti‐Mouse IgG (715‐025‐150, IF 1:160), and Fluorescein (FITC) AffiniPure Donkey Anti‐Mouse IgG (715‐095‐151, IF 1:160) used for immunostaining were purchased from Jackson Immuno Research (PA, USA).

### 4.2 Animal Models and Treatments

All the mouse strains were on a C57BL/6 background and kept in standard, infection‐free housing conditions, with 12 h light:12 h dark cycles and 3–5 mice per cage. Animals were housed in a pathogen‐free barrier environment throughout the study and fed a standard maintenance chow obtained from DOSSY Experimental Animals Co. Ltd. (China). This diet contains ≤ 10% moisture, ≥ 18% crude protein, ≥ 4% crude fat, ≤ 5% crude fiber, ≤ 8% total ash, 1.0%–1.8% calcium, 0.6%–1.2% phosphorus, 0.82% lysine, and 0.53% methionine + cystine. All animal experiments in this study were approved by the Institutional Animal Care and Use Committee (IACUC) of Sichuan University, and the procedures were performed according to the guidelines established by the China Council on Animal Care.

BK5.IGF‐1 transgenic (IGF‐1 Tg) and BK5.SIRT1 transgenic (SIRT1 Tg) mice were designed as previously described (DiGiovanni et al. 2000). Briefly, human IGF‐1 cDNA, encoding the prepro‐form of the IGF‐1 polypeptide, was inserted into the pRP(Exp) vector to generate BK5 > beta‐globin‐humanIGF‐1‐SV40 Poly A cassette. The mice were generated by Cyagen Biosciences (Suzhou, China), a highly reputable commercial company, which provided us with 3 male and 4 female founders derived from random insertion of the transgene. Seven transgenic founders were inbred for five generations, and two lines (CN and N) were selected for further study due to their stable hIGF‐1 expression, confirmed by RT‐PCR (Figure S2b). Specific primers used for genotyping are listed in Table S1.

To examine the effects of drug interventions: (Russell and Kahn 2007) Eight‐month IGF‐1 Tg mice or WT littermates were treated with Dasatinib (D, 5 mg/kg, Sigma, SML2589) + Quercetin (Q, 50 mg/kg, Sigma, Q4951) via oral gavage in the vehicle (10% ethanol + 30% polyethylene glycol 400 + 60% phosal 50 PG), at the frequency of once per day for 3 consecutive days followed by resting for 11 days. This was repeated for three cycles.

For DR experiments, 4‐month‐old IGF‐1 Tg mice or WT littermates were singly housed, and we recorded their average daily ad libitum food intake over 10 consecutive days. Subsequently, each mouse received 70% of its baseline intake for 6 months (i.e., a 30% reduction in total calories), while the nutrient composition remained unchanged.

As a positive control for apoptosis induced by DMBA (7,12‐dimethylbenz[a]anthracene, Selleck, E1022), mice were initiated with 25 nM DMBA in 0.2 mL of acetone, applied as a single topical application to the shaved dorsal skin of mice, and skin samples were collected two weeks post‐treatment.

### 4.3 Age‐Associated Gray Hair Quantification

The mice were placed in the center of the studio, and the digital images were captured with a Canon EOS7D Mark II. To quantify the degree of hair grayness, the digital images from the dorsal aspect of mice (2.7 cm × 2 cm) were subjected to ImageJ, a software used to analyze AOD as described (Ponnapakkam et al. 2015). Briefly, the images were converted to 8‐bit color depth using black hair for calibration. The AOD value of mice without white hair was set as 0 and increased with the grayness of the hair.

### 4.4 Human Skin Specimen

The use of human skin scalp tissues was reviewed and approved by the Ethics committee of West China Second University Hospital of Sichuan University, and samples were obtained after informed consent. Sections (5 μm) were cut from 4% paraformaldehyde (PFA)‐fixed samples for IHC.

### 4.5 Statistical Analyses

Student's *t*‐test was used for analyses that involved two groups for comparison, and ANOVA was used for analyses that involved more than two groups for comparisons.

### 4.6 Bulk RNA Sequencing Analysis

#### 4.6.1 Tissue Collection, Library Preparation, and Sequencing

For bulk RNAseq analysis, 10‐month‐old and 24‐month‐old wild‐type (WT) mice as well as 10‐month‐old IGF‐1 transgenic (IGF‐1 Tg) mice were shaved and sacrificed. Skin tissues from three mice in the same group were mixed with equal amounts and immediately homogenized in liquid nitrogen and stored on dry ice followed by further RNA sequencing analysis (OE Biotech Co. Ltd., Shanghai, China). Total RNA was extracted using TRIzol reagent (Invitrogen, CA, USA) according to the manufacturer's instructions. NanoDrop 2000 spectrophotometer (Thermo Scientific, USA) was used to assess the purity and quantity of RNA samples. RNA integrity was evaluated using the Agilent 2100 Bioanalyzer (Agilent Technologies, Santa Clara, CA, USA). VAHTS Universal V6 RNA‐seq Library Prep Kit was used to construct sequencing libraries according to the manufacturer's instructions. Then, 150 bp paired‐end transcriptome sequencing was performed on an Illumina Novaseq 6000 platform at OE Biotech Co. Ltd. (Shanghai, China).

#### 4.6.2 Quality Control, Differentially Expressed Genes, GSEA, and Visualization

We obtained about 46–49 million raw reads for each group. Low‐quality reads were then filtered out by fastp (Chen et al. 2018) and retained about 45–46 million clean reads per group. Clean reads were mapped to mm10 using HISAT2 (Kim et al. 2015). HTSeq‐count (Anders et al. 2015) was used to count the number of reads mapping to each gene. Non‐expressed genes were filtered out. Differential expression genes were identified using the DESeq2 package (Love et al. 2014). Gene set enrichment analysis was performed based on gene ranks by log2 fold change using the fgsea package (https://github.com/ctlab/fgsea). Normalized enrichment scores (NES score) of related pathways and corresponding genes were visualized using the pheatmap package (https://cran.r‐project.org/web/packages/pheatmap/).

### 4.7 Single‐Cell RNA Sequencing Analysis

#### 4.7.1 Tissue Collection, Single‐Cell Library Preparation, and Sequencing

For single‐cell RNA sequencing, 10‐month‐old WT and IGF‐1 Tg mice were shaved and sacrificed. Dorsal skins were then collected and enzymatically dissociated into a single‐cell suspension. The living cell fraction in each sample was estimated to be more than 90% and suitable for scRNA sequencing. Cells were then loaded onto the 10× Single Cell Chip G (10× Genomics) using Chromium Next GEM Single Cell 3' GEM, Library & Gel Bead Kit v3.1 (PN‐1000121, 10× Genomics) according to the manufacturer's protocol at OE Biotech Co. Ltd. (Shanghai, China). Briefly, the single‐cell sequencing library was constructed through GEM generation, barcoding, reverse transcription, cDNA amplification, fragmentation, end repair, A‐tailing, adaptor ligation, and size selection. The libraries were then sequenced on an Illumina NovaSeq X plus platform.

#### 4.7.2 Data Preprocessing, Integration, Clustering, and Cell‐Type Annotation

The fastq sequencing files were first processed with Cell Ranger (10× genomics) software to demultiplex cell barcodes, map reads to the mm10 genome and transcriptome. The resultant count matrix, features, and cell barcode files were then imported into the Seurat package (Hao et al. 2021). For each sample, we first removed low‐quality cells by the following criteria: (1) have less than 200 features or more than 6000 features; (2) more than 20% unique molecular identifiers (UMI) mapped to mitochondrial genes; or (3) more than 1% UMI mapped to hemoglobin genes. Then we used DoubletFinder (McGinnis et al. 2019) to remove potential doublets. After quality control steps, 8560 and 5879 cells remain in the WT and IGF‐1 Tg groups, respectively. Cells from the two groups were then integrated into one Seurat object using canonical correlation analysis implemented in the Seurat package. The integrated object then underwent dimension reduction, uniform manifold approximation and projection (UMAP), and clustering. Cells were then annotated into five major types: fibroblasts (Pdfga, Loxl1, and Fbln1), epidermis (Epcam, Krt14, and Krt5), immune cells (Ptprc), vascular cells (Tagln and Acta2) and neural‐crest‐derived cells (NCDC, Mbp, and Plp1). Fibroblasts are further annotated as telogen dermal papilla (tDP) and fibroblast types 1 to 4 subpopulations (Joost et al. 2020). Epidermis cells were further annotated as basal cells, supra, HFSCs, uHFs, niche, and sebaceous gland (SG) cells. NCDC cells were further annotated as Schwann cells, melanocytes, and lymph vessel cells. The differentially expressed genes between HFSCs from WT and IGF‐1 Tg mice were identified using the FindAllMarker function in the Seurat package. Gene ontology and reactome pathway enrichment analysis were carried out using the clusterProfiler package (Yu et al. 2012).

#### 4.7.3 Pseudotime Trajectory Analysis, Module Score Calculation, and Visualization

The monocle2 package (Qiu et al. 2017) was used to infer the pseudotime and trajectory of cells. Module score for a set of genes was calculated using the AddModuleScore function in the Seurat package. The significance of gene expression difference was determined by the Wilcoxon rank‐sum test. The significance of module score differences between the two groups was determined by the student's t test. The UMAP plots, violin plots, and dot plots were generated using DimPlot, VlnPlot, and DotPlot functions, respectively. Cell trajectory plots were generated using the plot_cell_trajectory function in the monocle2 package. The bar plots were generated using the ggplot2 package.

## Author Contributions

Z.‐X.J.X. and Y.W. conceived and designed the study. Y.W., Yue L., and Z.Z. conducted most of the animal experiments, IHC, IF, biochemical, and molecular experiments, with support from J.L., Y.P., L.W., Y.Y., M.X., M.W., J.L., and W.L., J.Y. and Y.Y. carried out RNA‐seq and single‐cell sequencing analyses. Y.Z., Y.Y., Y.C., Y.L., and Q.L. contributed to data interpretation and discussion. J.Z. and Q.L. were responsible for the collection of human skin samples. Z.‐X.J.X., Y.W., and Y.Z. drafted the manuscript. All authors reviewed and approved the final manuscript.

## Conflicts of Interest

Patents on senolytic drugs and their uses are held by Mayo Clinic. This research has been reviewed by the Mayo Clinic Conflict of Interest Review Board and was conducted in compliance with Mayo Clinic Conflict of Interest policies.

## Supporting information


**Figure S1.** Analysis of IGF‐1 expression with age in various organs using the Tabula Muris Senis database. (https://twc‐stanford.shinyapps.io/maca/, Access date 20240618).

**Figure S2.** (a) Schematic representation of the BK5.IGF‐1 transgenic (IGF‐1 Tg) cassette, consisting of a bovine keratin 5 (BK5) promoter, rabbit β‐globin intron, human IGF‐1 cDNA, and SV40 polyadenylation signal sequence (DiGiovanni et al. 2000). Primers (F and R) used for genotyping IGF‐1 Tg mice are indicated. (b) Representative gel image from RT‐PCR analysis showing hIGF‐1 expression in skin samples from IGF‐1 Tg mice and WT littermates. (c and d) Immunohistochemical (IHC) analyses of human IGF‐1 expression in major organs of IGF‐1 Tg or WT mice. Quantification was carried out by average optical density (AOD) (4‐month, male, *n* = 2/group). Scale bar = 50 μm. (e and f) The plasma levels of human IGF‐1 (hIGF‐1), mouse IGF‐1 (mIGF‐1) (e), insulin, and growth hormone (GH) (f) in peripheral blood of IGF‐1 Tg or non‐transgenic littermates (WT) (2‐, 10‐month, male, *n* = 5/group) were examined by ELISA. Data were means ± SEM, Two‐way ANOVA with Tukey’s test.

**Figure S3.** (a) Monthly body weights of IGF‐1 (red) and WT (blue) mice (male, *n* = 8/group). (b) Representative images of hair coats from 10‐month‐old IGF‐1 Tg mice, 10‐month‐old WT mice, and 24‐month‐old WT mice, with arrowheads highlighting areas of hair loss. (c and d) 2‐month‐old IGF‐1 Tg male mice and WT male littermates were shaved and monitored for hair coat recovery. Quantifications represent the percentage of back skin covered by regrown hair. Data are presented as means ± SD, *n* = 5/group. Two‐tailed Student’s *t*‐test.

**Figure S4.** (a and b). Dorsal skin samples were collected from 10‐ and 24‐month‐old WT mice and 10‐month‐old IGF‐1 Tg male mice (*n* = 3/group). Tissues from each group were pooled in equal amounts and subjected to RNA‐seq transcriptome analyses. Gene set enrichment analysis (GSEA) identified altered signaling pathways related to skin development and immune responses, shown as normalized enrichment scores (NES) (a). The heatmap (b) displays the expression levels of representative genes in each pathway. (c) Histological examination of dorsal skin from 10‐month‐old IGF‐1 Tg mice, 10‐month‐old WT mice, and 24‐month‐old WT mice. Representative IHC images and corresponding quantitative analyses for p53, cellular senescence markers p16 and PAI‐1 are shown. Data are presented as means ± SEM, *n* = 5/group. Two‐way ANOVA with Tukey’s test. Scale bar = 50 μm.

**Figure S5.** (a) Dorsal skin samples were obtained from 10‐month‐old WT and IGF‐1 Tg male mice, enzymatically digested into single cells, and analyzed using single‐cell RNA sequencing. UMAP was plotted to display the distribution of five principal cell types. UMAP plots show the expression of *Igf1* in skin cells from WT and IGF‐1 Tg mice. (b) Violin plot illustrating representative markers used to identify the five main cell types. (c) Among the five major cell types identified, epidermal cells were selected for further clustering. The dot plot shows the expression of representative markers used to distinguish subtypes of epidermal cells. (d) The percentage of each major cell type in IGF‐1 Tg and WT mice is displayed. (e) Pseudotime trajectory analysis of HFSC and Upper HF. The upper plots display pseudotime and cell types along the trajectory. The lower plots illustrate cell distributions along the trajectory for WT and IGF‐1 Tg mice, respectively. (f) Violin plots display the expression levels of *Jag1* and *Tgfb1* in HFSCs. Wilcoxon rank‐sum test.

**Figure S6.** (a) UMAP plot illustrating subtypes of neural‐crest‐derived cells (NCDC) in skin samples from WT and IGF‐1 Tg mice. (b) Dot plot showing the expression of representative markers used to identify subtypes of NCDC.

**Figure S7.** TUNEL staining of dorsal skin from 10‐month‐old IGF‐1 Tg male mice and their WT male littermates (*n* = 5/group). Dorsal skin of 10‐month‐old WT mice treated with DMBA was used as a positive control for TUNEL staining.

**Figure S8.** (a) UMAP plot depicting fibroblast subtypes in skin samples from WT and IGF‐1 Tg mice. (b) Dot plot depicting the expression of unique markers used to identify subtypes of fibroblasts. (c) Bar plot displaying the composition of fibroblast subtypes between WT and IGF‐1 Tg mice. (d) Violin plots illustrating the expression differences in senescence‐associated genes (*Cdkn1a, Il6*, *cxcl2*) in dermal papilla (DP) cells. Wilcoxon rank‐sum test. (e) Senescence‐associated beta‐galactosidase (SA‐β‐gal) staining conducted on dorsal skin from 10‐month‐old IGF‐1 Tg mice, 10‐month‐old WT mice, and 24‐month‐old WT mice. (f) DP cells isolated from 10‐month‐old IGF‐1 transgenic mice and age‐matched WT littermates were cultured and subjected to SA‐β‐gal staining to assess senescence.

**Figure S9.** (a–c) Violin plot for cell cycle arrest gene p21 (*Cdkn1a*) and collagens (*Col1a1* and *Col1a2*) in subtypes of fibroblasts from WT and IGF‐1 Tg mice. Wilcoxon rank‐sum test was used to determine the significance of expression differences. (d) Masson staining for the collagen and quantification of dermal thickness were detected in IGF‐1 Tg mice and WT littermates. *n* = 10/group. Data were means ± SEM. Statistical analysis was performed using two‐tailed Student’s *t*‐test. Scale bar = 100 μm. (e) The network diagram illustrates the interactions and strengths of IGF‐1 signaling among various skin cell types. edge weights are proportional to the interaction strength.

**Figure S10.** (a) Schematic representation of BK5.SIRT1 transgenic (SIRT1 Tg) mice. (b) RT‐PCR analyses were performed to examine SIRT1 mRNA expression in the liver, kidney or skin of 4 m SIRT1‐TG mice (N and T strains) and WT mice. (c) Genotyping of BK5.SIRT1 transgenic (SIRT1 Tg) mice, similar to methods outlined in Figure S2a, utilizing mouse SIRT1 cDNA.

**Table S1.** Specific primers used for genotyping are listed.

## Data Availability

The raw single‐cell and bulk RNA sequencing data of mouse skin have been deposited in the NCBI Sequence Read Archive (SRA) under accession numbers PRJNA1240205 and PRJNA1240226, respectively. The data that supports the findings of this study are available in the Supporting Information of this article.
