# Supplementary figures and images for: Targeting IGF1‐Induced Cellular Senescence to Rejuvenate Hair Follicle Aging
Source: Aging Cell. 2025 Mar 30;24(7):e70053. doi: 10.1111/acel.70053 (PMC12266755; doi:10.1111/acel.70053)

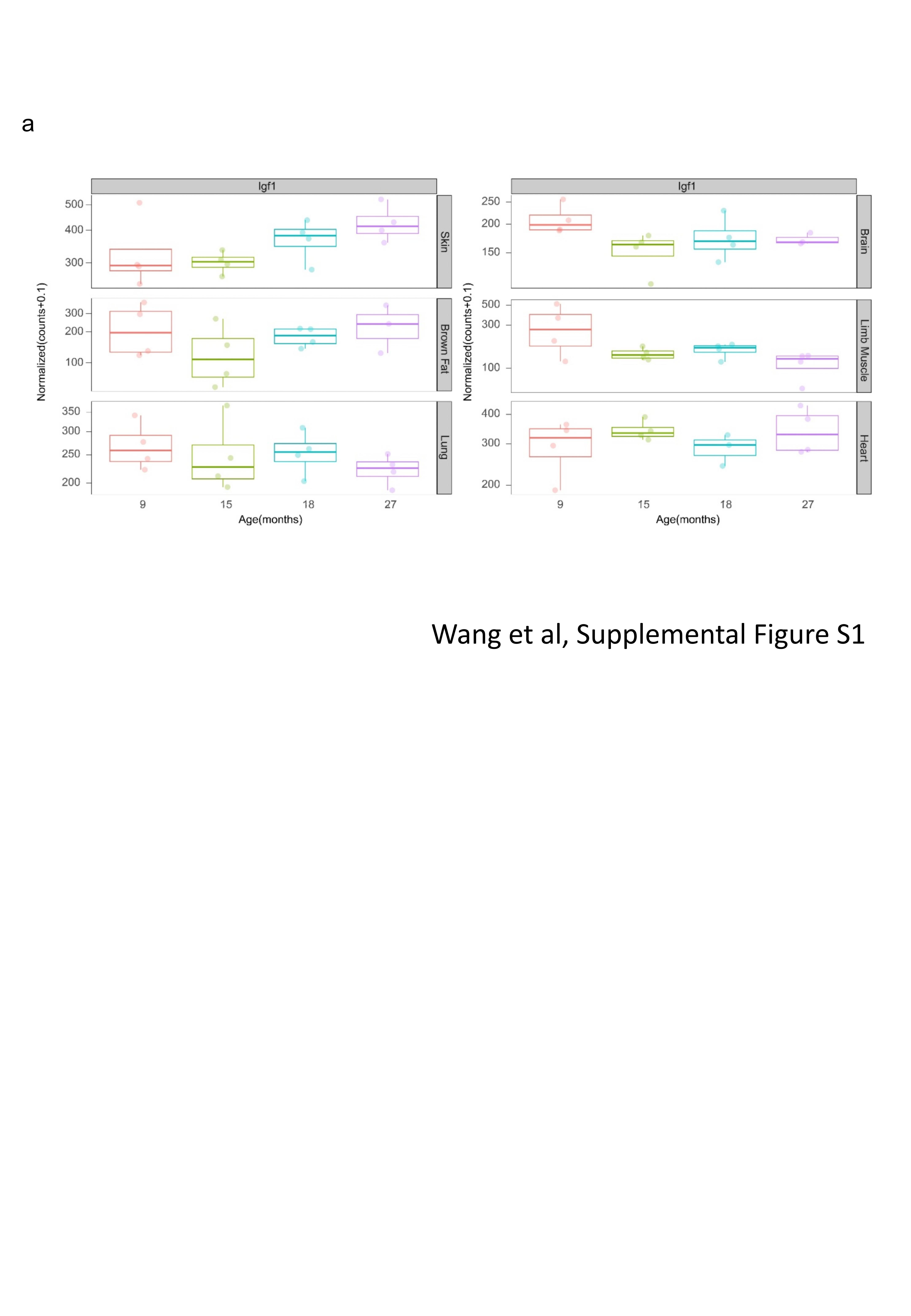

Supplement: Supplementary file 1 — Figure S1. Analysis of IGF‐1 expression with age in various organs using the Tabula Muris Senis database. (https://twc‐stanford.shinyapps.io/maca/, Access date 20240618). [file ACEL-24-e70053-s004.jpg]

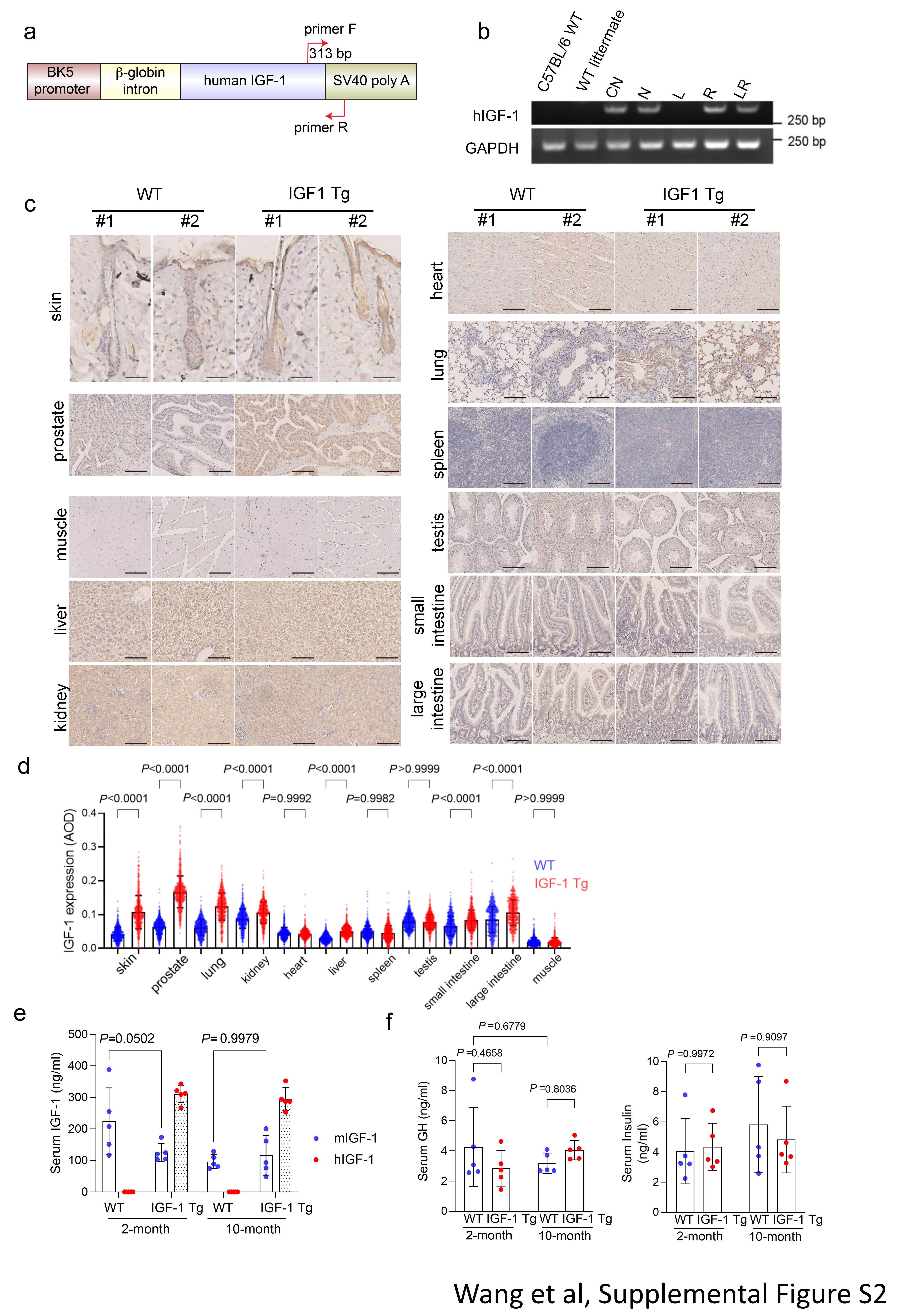

Supplement: Supplementary file 2 — Figure S2. (a) Schematic representation of the BK5.IGF‐1 transgenic (IGF‐1 Tg) cassette, consisting of a bovine keratin 5 (BK5) promoter, rabbit β‐globin intron, human IGF‐1 cDNA, and SV40 polyadenylation signal sequence (DiGiovanni et al. 2000). Primers (F and R) used for genotyping IGF‐1 Tg mice are indicated. (b) Representative gel image from RT‐PCR analysis showing hIGF‐1 expression in skin samples from IGF‐1 Tg mice and WT littermates. (c and d) Immunohistochemical (IHC) analyses of human IGF‐1 expression in major organs of IGF‐1 Tg or WT mice. Quantification was carried out by average optical density (AOD) (4‐month, male, n = 2/group). Scale bar = 50 μm. (e and f) The plasma levels of human IGF‐1 (hIGF‐1), mouse IGF‐1 (mIGF‐1) (e), insulin, and growth hormone (GH) (f) in peripheral blood of IGF‐1 Tg or non‐transgenic littermates (WT) (2‐, 10‐month, male, n = 5/group) were examined by ELISA. Data were means ± SEM, Two‐way ANOVA with Tukey’s test. [file ACEL-24-e70053-s005.jpg]

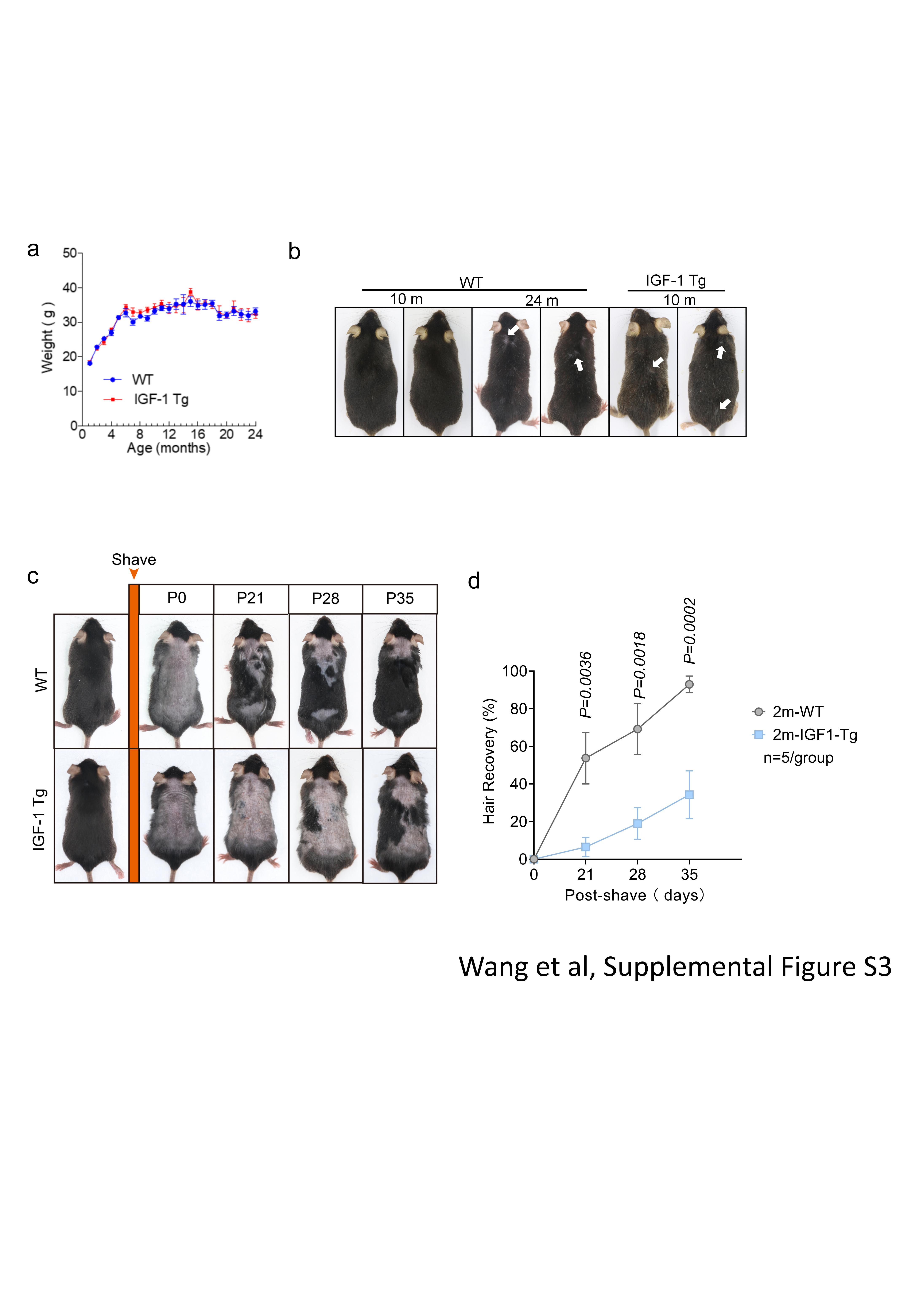

Supplement: Supplementary file 3 — Figure S3. (a) Monthly body weights of IGF‐1 (red) and WT (blue) mice (male, n = 8/group). (b) Representative images of hair coats from 10‐month‐old IGF‐1 Tg mice, 10‐month‐old WT mice, and 24‐month‐old WT mice, with arrowheads highlighting areas of hair loss. (c and d) 2‐month‐old IGF‐1 Tg male mice and WT male littermates were shaved and monitored for hair coat recovery. Quantifications represent the percentage of back skin covered by regrown hair. Data are presented as means ± SD, n = 5/group. Two‐tailed Student’s t‐test. [file ACEL-24-e70053-s007.jpg]

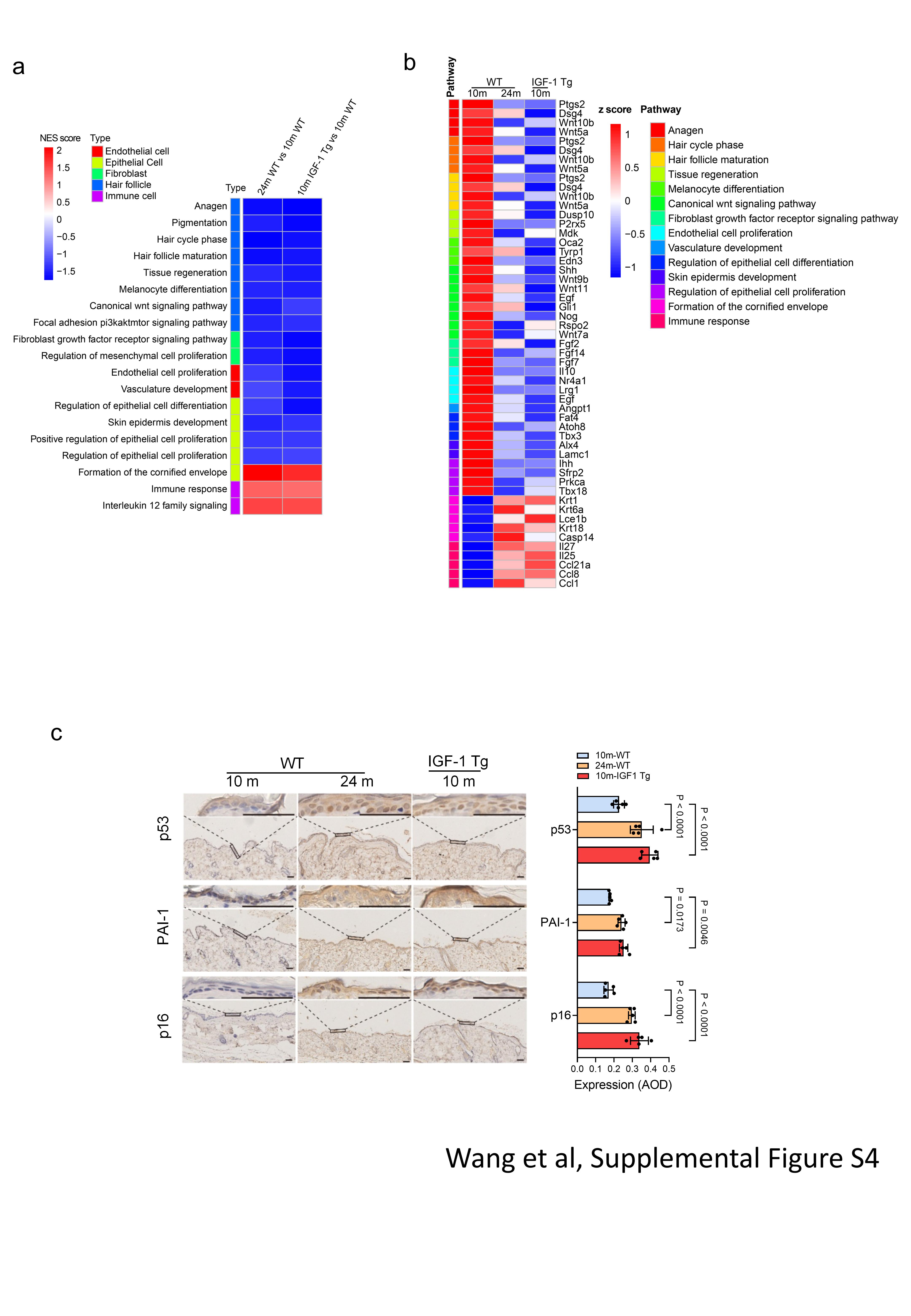

Supplement: Supplementary file 4 — Figure S4. (a and b). Dorsal skin samples were collected from 10‐ and 24‐month‐old WT mice and 10‐month‐old IGF‐1 Tg male mice (n = 3/group). Tissues from each group were pooled in equal amounts and subjected to RNA‐seq transcriptome analyses. Gene set enrichment analysis (GSEA) identified altered signaling pathways related to skin development and immune responses, shown as normalized enrichment scores (NES) (a). The heatmap (b) displays the expression levels of representative genes in each pathway. (c) Histological examination of dorsal skin from 10‐month‐old IGF‐1 Tg mice, 10‐month‐old WT mice, and 24‐month‐old WT mice. Representative IHC images and corresponding quantitative analyses for p53, cellular senescence markers p16 and PAI‐1 are shown. Data are presented as means ± SEM, n = 5/group. Two‐way ANOVA with Tukey’s test. Scale bar = 50 μm. [file ACEL-24-e70053-s009.jpg]

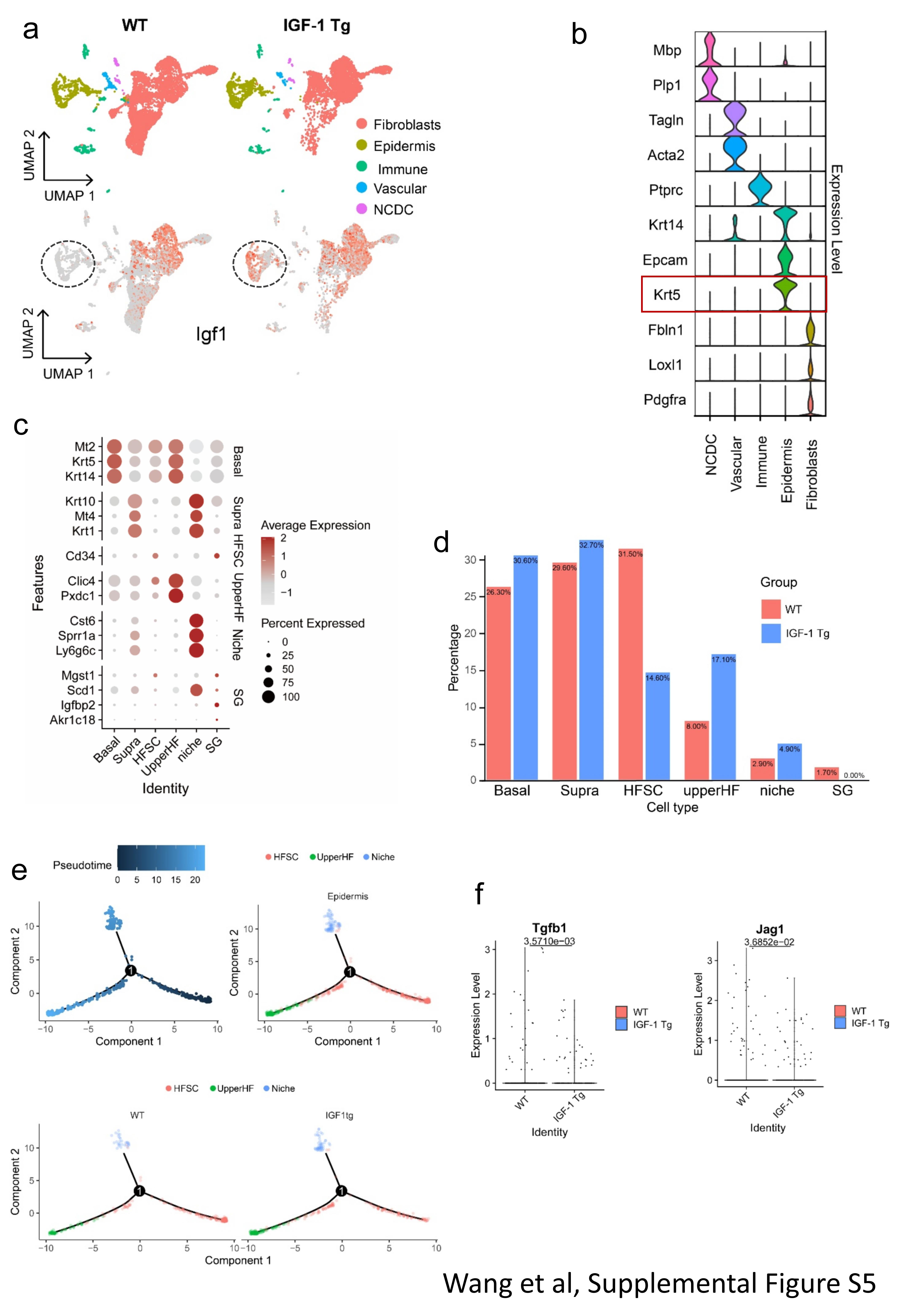

Supplement: Supplementary file 5 — Figure S5. (a) Dorsal skin samples were obtained from 10‐month‐old WT and IGF‐1 Tg male mice, enzymatically digested into single cells, and analyzed using single‐cell RNA sequencing. UMAP was plotted to display the distribution of five principal cell types. UMAP plots show the expression of Igf1 in skin cells from WT and IGF‐1 Tg mice. (b) Violin plot illustrating representative markers used to identify the five main cell types. (c) Among the five major cell types identified, epidermal cells were selected for further clustering. The dot plot shows the expression of representative markers used to distinguish subtypes of epidermal cells. (d) The percentage of each major cell type in IGF‐1 Tg and WT mice is displayed. (e) Pseudotime trajectory analysis of HFSC and Upper HF. The upper plots display pseudotime and cell types along the trajectory. The lower plots illustrate cell distributions along the trajectory for WT and IGF‐1 Tg mice, respectively. (f) Violin plots display the expression levels of Jag1 and Tgfb1 in HFSCs. Wilcoxon rank‐sum test. [file ACEL-24-e70053-s002.jpg]

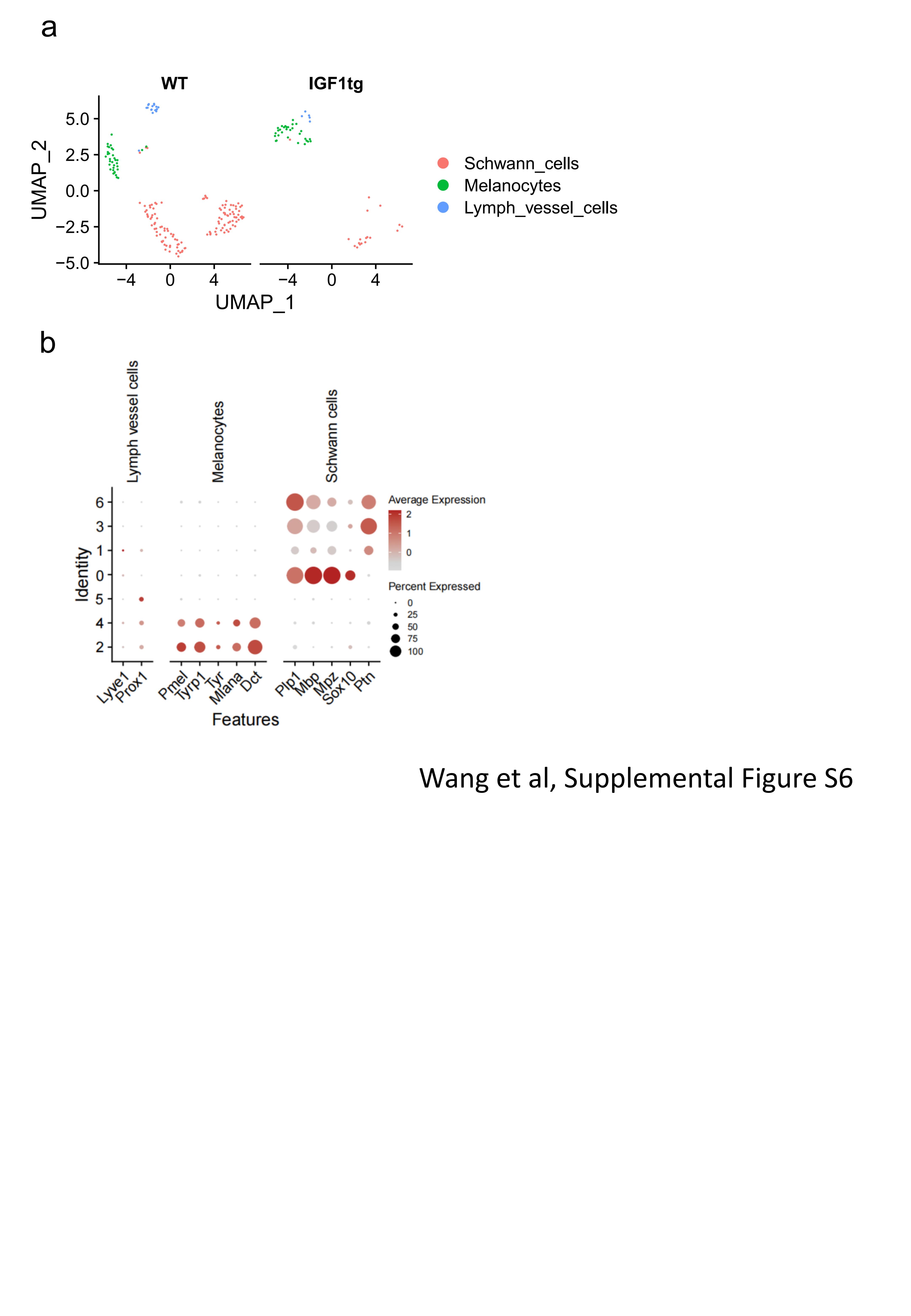

Supplement: Supplementary file 6 — Figure S6. (a) UMAP plot illustrating subtypes of neural‐crest‐derived cells (NCDC) in skin samples from WT and IGF‐1 Tg mice. (b) Dot plot showing the expression of representative markers used to identify subtypes of NCDC. [file ACEL-24-e70053-s001.jpg]

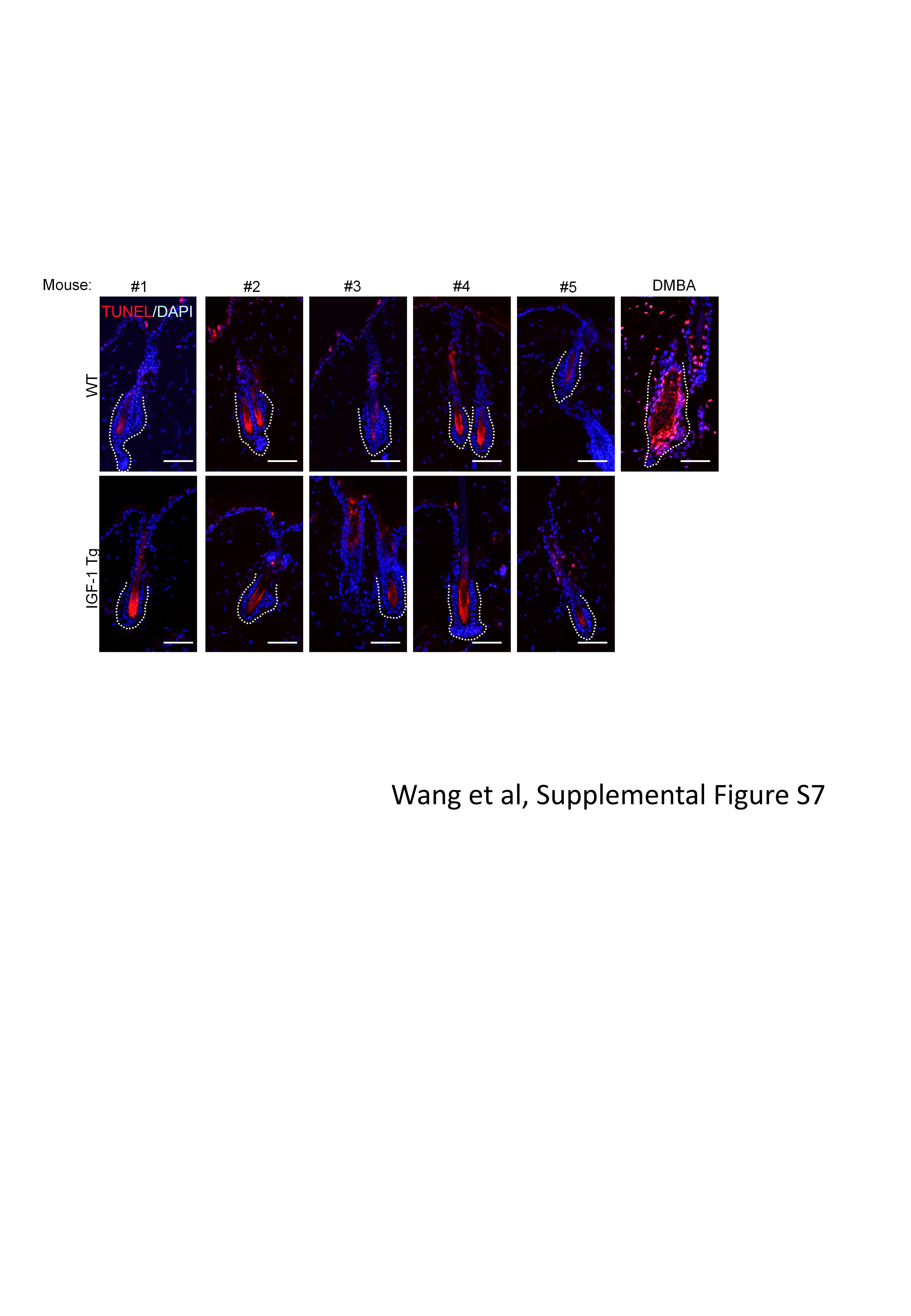

Supplement: Supplementary file 7 — Figure S7. TUNEL staining of dorsal skin from 10‐month‐old IGF‐1 Tg male mice and their WT male littermates (n = 5/group). Dorsal skin of 10‐month‐old WT mice treated with DMBA was used as a positive control for TUNEL staining. [file ACEL-24-e70053-s010.jpg]

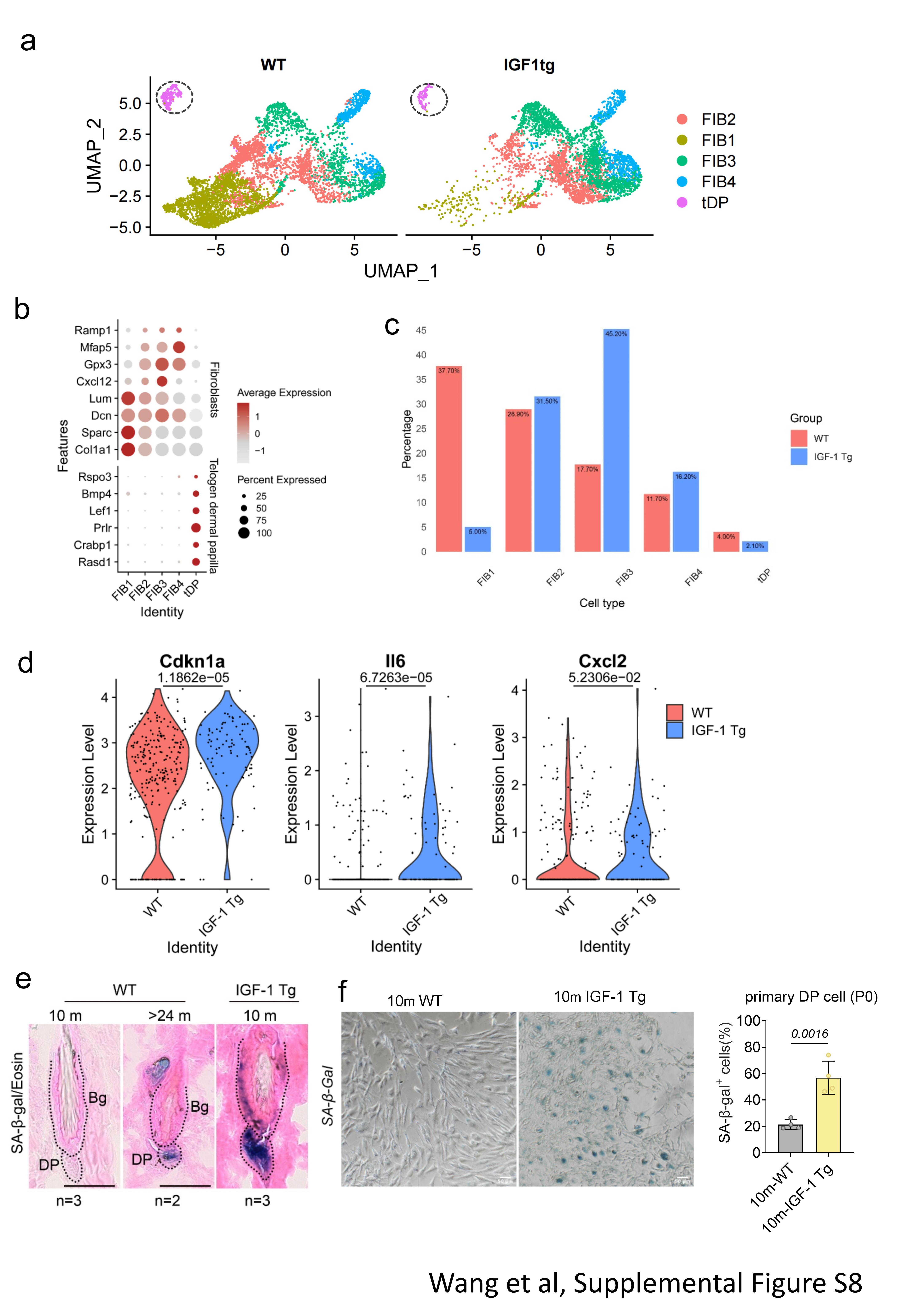

Supplement: Supplementary file 8 — Figure S8. (a) UMAP plot depicting fibroblast subtypes in skin samples from WT and IGF‐1 Tg mice. (b) Dot plot depicting the expression of unique markers used to identify subtypes of fibroblasts. (c) Bar plot displaying the composition of fibroblast subtypes between WT and IGF‐1 Tg mice. (d) Violin plots illustrating the expression differences in senescence‐associated genes (Cdkn1a, Il6, cxcl2) in dermal papilla (DP) cells. Wilcoxon rank‐sum test. (e) Senescence‐associated beta‐galactosidase (SA‐β‐gal) staining conducted on dorsal skin from 10‐month‐old IGF‐1 Tg mice, 10‐month‐old WT mice, and 24‐month‐old WT mice. (f) DP cells isolated from 10‐month‐old IGF‐1 transgenic mice and age‐matched WT littermates were cultured and subjected to SA‐β‐gal staining to assess senescence. [file ACEL-24-e70053-s008.jpg]

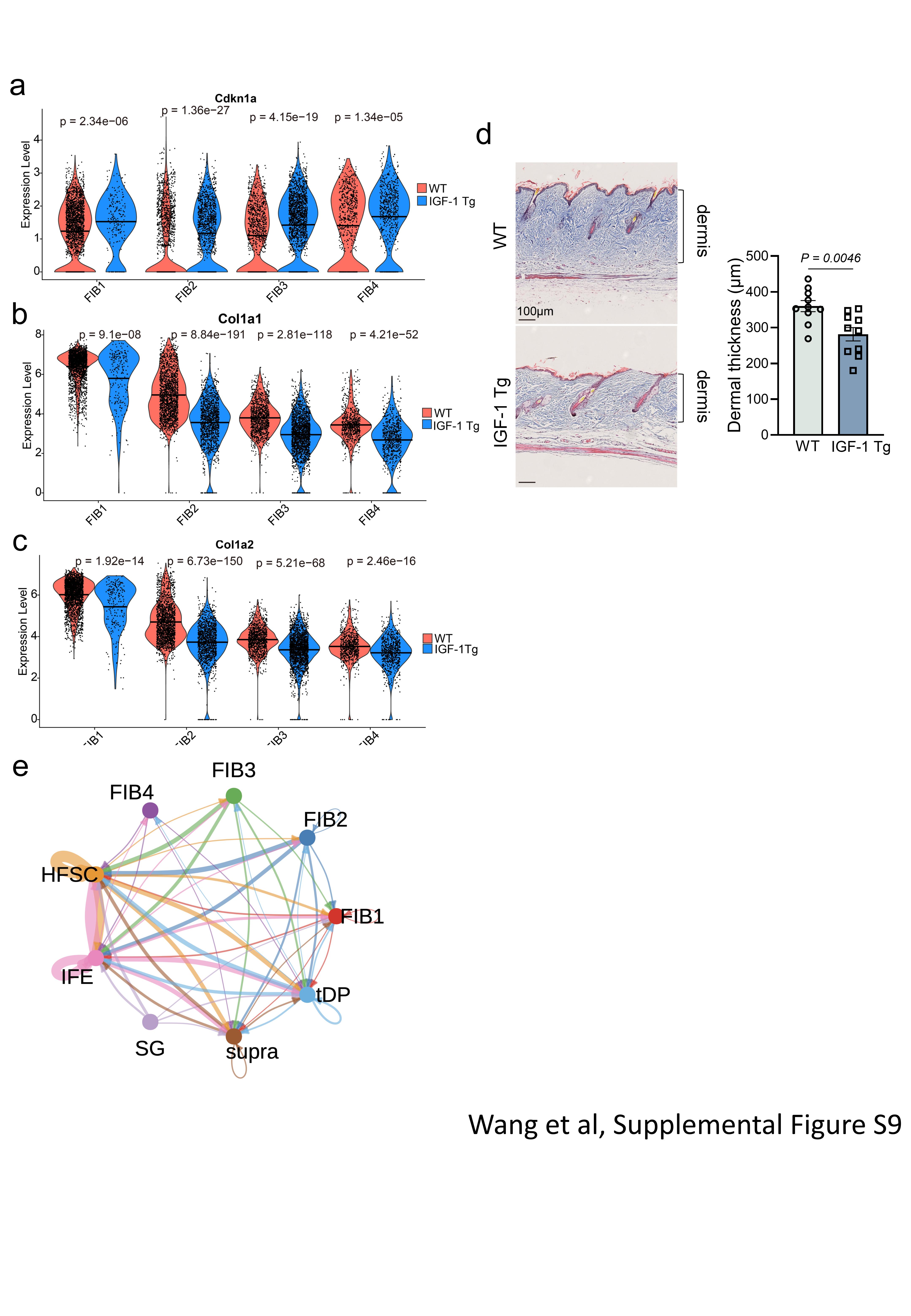

Supplement: Supplementary file 9 — Figure S9. (a–c) Violin plot for cell cycle arrest gene p21 (Cdkn1a) and collagens (Col1a1 and Col1a2) in subtypes of fibroblasts from WT and IGF‐1 Tg mice. Wilcoxon rank‐sum test was used to determine the significance of expression differences. (d) Masson staining for the collagen and quantification of dermal thickness were detected in IGF‐1 Tg mice and WT littermates. n = 10/group. Data were means ± SEM. Statistical analysis was performed using two‐tailed Student’s t‐test. Scale bar = 100 μm. (e) The network diagram illustrates the interactions and strengths of IGF‐1 signaling among various skin cell types. edge weights are proportional to the interaction strength. [file ACEL-24-e70053-s003.jpg]

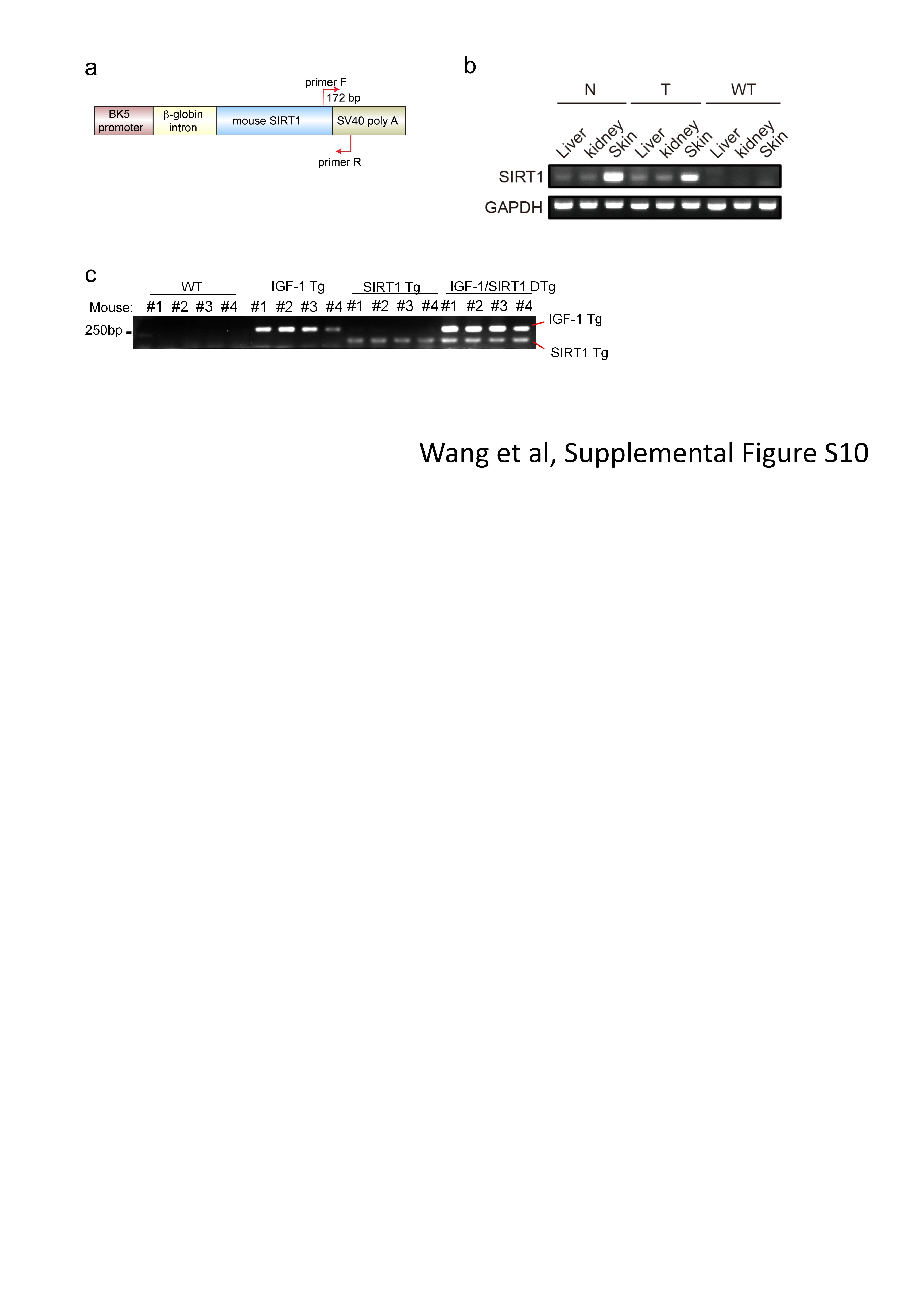

Supplement: Supplementary file 10 — Figure S10. (a) Schematic representation of BK5.SIRT1 transgenic (SIRT1 Tg) mice. (b) RT‐PCR analyses were performed to examine SIRT1 mRNA expression in the liver, kidney or skin of 4 m SIRT1‐TG mice (N and T strains) and WT mice. (c) Genotyping of BK5.SIRT1 transgenic (SIRT1 Tg) mice, similar to methods outlined in Figure S2a, utilizing mouse SIRT1 cDNA. [file ACEL-24-e70053-s006.jpg]
