# Supplementary material for: Targeting IGF1‐Induced Cellular Senescence to Rejuvenate Hair Follicle Aging
Source: Aging Cell. 2025 Mar 30;24(7):e70053. doi: 10.1111/acel.70053 (PMC12266755; doi:10.1111/acel.70053)
Supplement: Supplementary file 11 — Table S1. Specific primers used for genotyping are listed. [file ACEL-24-e70053-s011.pdf]

Supplemental Table S1

IGF-1 Tg mice	F 5-CCACAGGGTATGGCTCC-3	R 5-GTCCAAACTCATCAATGTATCTT-3
SIRT1 Tg mice	F 5-TTGGAAGATGATACGGAGAGG-3	R 5-GCCATCACCACTTTGTACAAG-3

Supplemental Table S1. Specific primers used for genotyping are listed.
